# Photochemistry of Ru(II) Triazole Complexes with 6-Membered Chelate Ligands: Detection and Reactivity of Ligand-Loss Intermediates

**DOI:** 10.1021/acs.inorgchem.4c00251

**Published:** 2024-05-03

**Authors:** Katie Eastham, Aaron D. W. Kennedy, Synøve Ø. Scottwell, Jack E. Bramham, Samantha Hardman, Alexander P. Golovanov, Paul A. Scattergood, James D. Crowley, Paul I. P. Elliott

**Affiliations:** † Department of Chemical Sciences and Centre for Functional Materials, University of Huddersfield, Queensgate, Huddersfield HD1 3DH, U.K.; ‡ Department of Chemistry, University of Otago, PO Box 56, Dunedin 9054, New Zealand; § MacDiarmid Institute for Advanced Materials and Nanotechnology, Wellington 6140, New Zealand; ∥ Manchester Institute of Biotechnology, The University of Manchester, 131 Princess Street, Manchester M1 7DN, U.K.; ⊥ Department of Chemistry, School of Natural Sciences, Faculty of Science and Engineering, The University of Manchester, Manchester M13 9PL, U.K.

## Abstract

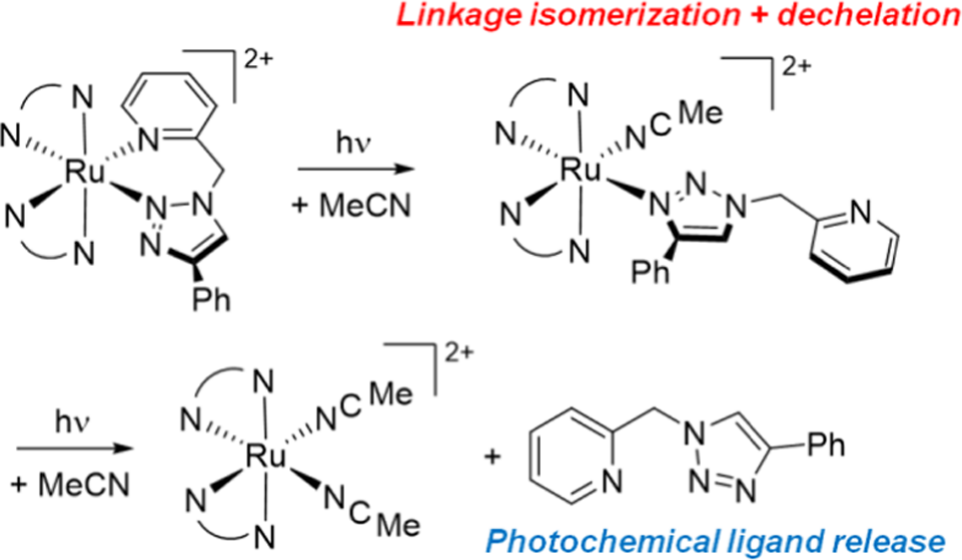

Photochemical ligand release from metal complexes may be exploited in the development of novel photoactivated chemotherapy agents for the treatment of cancer and other diseases. Highly intriguing photochemical behavior is reported for two ruthenium(II) complexes bearing conformationally flexible 1,2,3-triazole-based ligands incorporating a methylene spacer to form 6-membered chelate rings. [Ru(bpy)_2_(pictz)]^2+^ (**1**) and [Ru(bpy)_2_(btzm)]^2+^ (**2**) (bpy = 2,2′-bipyridyl; pictz = 1-(picolyl)-4-phenyl-1,2,3-triazole; btzm = bis(4-phenyl-1,2,3-triazol-4-yl)methane) exhibit coordination by the triazole ring through the less basic N2 atom as a consequence of chelation and readily undergo photochemical release of the pictz and btzm ligands (ϕ = 0.079 and 0.091, respectively) in acetonitrile solution to form *cis*-[Ru(bpy)_2_(NCMe)_2_]^2+^ (**3**) in both cases. Ligand-loss intermediates of the form [Ru(bpy)_2_(κ^1^-pictz or κ^1^-btzm)(NCCD_3_)]^2+^ are detected by ^1^H NMR spectroscopy and mass spectrometry. Photolysis of **1** yields three ligand-loss intermediates with monodentate pictz ligands, two of which form through simple decoordination of either the pyridine or triazole donor with subsequent solvent coordination (**4-tz**^**(N2)**^ and **4-py**, respectively). The third intermediate, shown to be able to form photochemically directly from **1**, arises through linkage isomerism in which the monodentate pictz ligand is coordinated by the triazole N3 atom (**4-tz**^**(N3)**^) with a comparable ligand-loss intermediate with an N3-bound κ^1^-btzm ligand also observed for **2**.

## Abstract 2

Ruthenium(II) complexes with chelating triazole-based ligands in which coordination via the N2-triazole undergoes intriguing photochemical ligand-release reactivity involving monodentate solvento intermediates which form through both ligand dechelation and linkage isomerism, resulting in the coordination of the ligand through the more favorable triazole N3 atom. The paper details our studies by UV–visible and NMR spectroscopy on this unusual photochemical reactivity.

## Introduction

The attractive photophysical properties of kinetically inert oligopyridyl transition metal complexes^1^ have seen their use in various applications including light-emitting devices,^2^ dye-sensitized solar cells,^3^ photocatalytic sensitization,^4,5^ confocal luminescence imaging microscopy,^6,7^ and for therapeutic use in photodynamic therapy.^8,9^ The successful exploitation of these materials is dependent on the relatively long-lived triplet metal-to-ligand charge transfer (^3^MLCT) states, which are responsible for luminescent emission and excited-state electron/energy transfer reactivity. These ^3^MLCT states can under certain circumstances undergo rapid deactivation through population of metal-centered (^3^MC) states.^10−13^ These ^3^MC states, involving significant structural distortions, are the precursor to photochemical reactivity whereby ligands can undergo dechelation leading to isomerization or formal ligand dissociation.^14−20^ While deleterious for photocatalytic or device applications, the deliberate engineering of ^3^MC state population into complexes through appropriate structural design enables application in light-activated drug release in photoactivated chemotherapy (PACT).^21−25^

**Figure 1:**
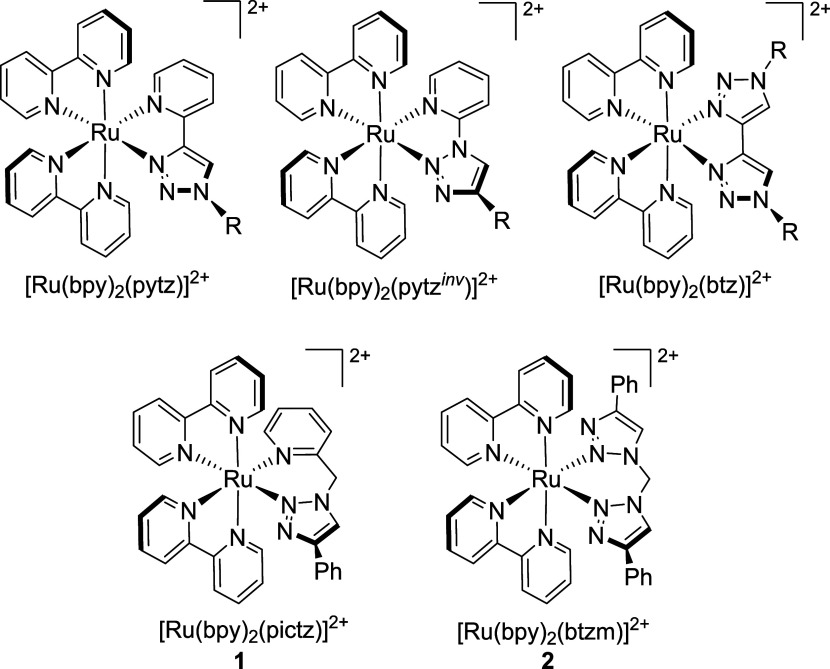
Structures of previously reported photoreactive triazole-containing complexes [Ru(bpy)_2_(pytz)]^2+^, [Ru(bpy)_2_(pytz^inv^)]^2+^, and [Ru(bpy)_2_(btz)]^2+^, as well as the new complexes [Ru(bpy)_2_(pictz)]^2+^ (**1**) and [Ru(bpy)_2_(btzm)]^2+^ (**2**) reported here.

Promotion of photochemical reactivity can be achieved through incorporation of steric congestion onto the ligands such that the metal–ligand bonds are weakened, thus stabilizing ^3^MC states and resulting in their rapid population from photoexcited ^3^MLCT states.^21,26−29^ Such steric promotion has also enabled the direct experimental spectroscopic evidence for ^3^MC states.^30−32^ We have previously reported the surprisingly facile photochemical chelate ligand release in the absence of any steric promotion for complexes bearing triazole-containing ligands. The heteroleptic complexes [Ru(bpy)_2_(btz)]^2+^ (Figure 1) and [Ru(bpy)(btz)_2_]^2+^ (bpy = 2,2′-bipyridyl, btz = 1,1′-dibenzyl-4,4′-bi-1,2,3-triazolyl) undergo photochemical dissociation of a btz ligand.^33−35^ The btz lowest unoccupied molecular orbital (LUMO) is significantly higher in energy than that of bpy leading to destabilization of the bpy-centered ^3^MLCT states of these complexes,^36^ which promotes ^3^MC state population and thus photochemical reactivity.^16^ The electronic effects imparted by these ligands also led to the unprecedented observation of photochemical ligand ejection from an osmium(II) complex, [Os(btz)_3_]^2+^.^37^

In the vast majority of 1,2,3-triazole-based complexes coordination to the metal occurs through the more basic N3-position^38,39^ but can be induced to coordinate through the N2 atom through chelation.^40−42^ We have shown that while the 4-(pyrid-2-yl)-1,2,3-triazole (pytz) complex [Ru(bpy)_2_(pytz)]^2+^ (Figure 1) is only moderately photochemically reactive, the corresponding complex of the inverted pytz ligand 1-(pyrid-2-yl)-1,2,3-triazole (pytz^inv^) is far more photochemically reactive.^43^ This prompted us to further investigate the photochemistry of complexes in which triazole donors are constrained to coordinate through the N2 atom of the triazole ring.

In this contribution we report the synthesis, characterization, and photochemical reactivity of complexes of the ligands 1-(2-picolyl)-4-phenyl-1,2,3-triazole (pictz) and bis(4-phenyl-1,2,3-triazol-1-yl)methane (btzm) in which the triazole-containing ligands additionally contain a methylene bridge between their donor heterocycles thus resulting in more flexible 6-membered chelate rings (Figure 1). Here, we envisaged that the expanded chelate ring formed by these ligands would confer greater excited-state conformational freedom, which might result in increased photochemical reactivity which has been shown with other systems that feature diamine and thioether-based departing ligands that form 6-membered chelate rings.^25,44−50^ We show that these complexes indeed undergo relatively efficient photochemical ejection of the triazole-containing ligand and provide spectroscopic evidence of the formation of monodentate ligand-loss solvent complex intermediates. Further, we show that in these monodentate complexes, linkage isomerism can be induced whereby the triazole-containing ligand converts from the initial coordination mode via the N2-position to become coordinated by the more basic N3 atom.

## Results and Discussion

### Synthesis and Characterization

The ligand pictz was prepared by a method that we have reported previously.^51^ The symmetrical ligand btzm was prepared through microwave-assisted copper-catalyzed cycloaddition of phenylacetylene with diazidomethane in dimethylformamide exploiting a method that we have used previously for the synthesis of related 1,2,3-triazole ligands.^52^**WARNING!** Low-molecular-weight azides, and diazides, are potentially explosive and should never be isolated, but only generated in situ! Thus, the highly reactive diazidomethane is not isolated but is prepared through reaction of sodium azide with dichloromethane and is subsequently consumed through cycloaddition with phenylacetylene. Complexes **1** and **2** were then prepared through the reaction of [Ru(bpy)_2_Cl_2_] with the ligands pictz and btzm, respectively, with microwave irradiation and were isolated as their hexafluorophosphate salts.

**Figure 2:**
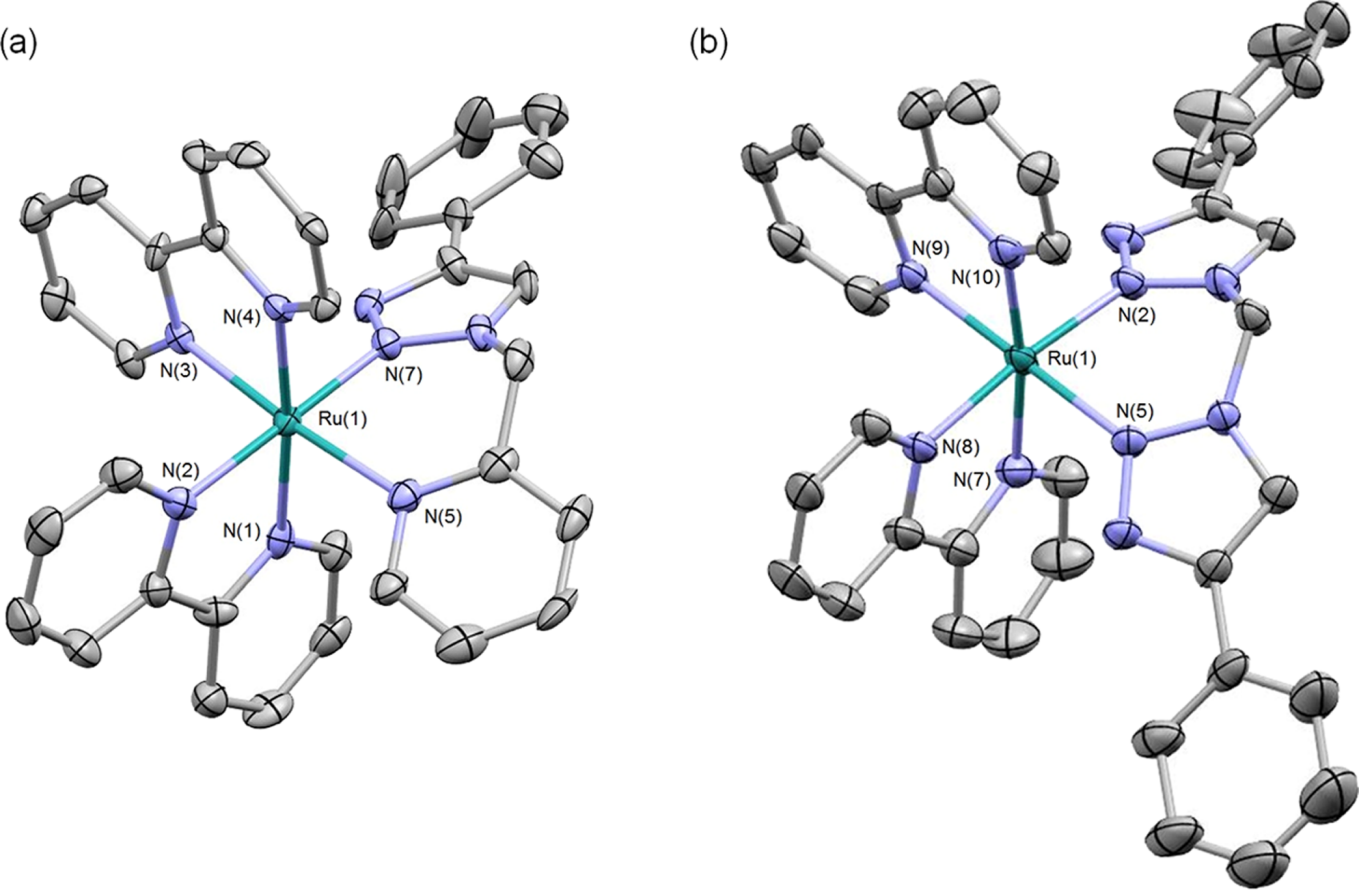
Structures of the cations for **1** (a) and **2** (b) from X-ray crystallographic analysis (ellipsoids at 50% probability). Hydrogen atoms, cocrystallized solvent, and counterions were removed for clarity. Selected bond lengths (Å) and angles (deg): For **1**, Ru(1)–N(1) 2.079(9); Ru(1)–N(2) 2.051(8); Ru(1)–N(3) 2.035(8); Ru(1)–N(4) 2.072(8); Ru(1)–N(5) 2.122(9); Ru(1)–N(7) 2.056(9); N(7)–Ru(1)–N(5) 89.1(4); N(1)–Ru(1)–N(2) 79.2(3); N(3)–Ru(1)–N(4) 79.2(3); N(1)–Ru(1)–N(4) 173.7(3); N(2)–Ru(1)–N(7) 172.1(3); N(3)–Ru(1)–N(5) 175.0(3). For, **2** Ru(1)–N(2) 2.086(4); Ru(1)–N(5) 2.067(4); Ru(1)–N(7) 2.067(5); Ru(1)–N(8) 2.053(5); Ru(1)–N(9) 2.055(5); Ru(1)–N(10) 2.080(5); N(2)–Ru(1)–N(5) 88.05(18); N(7)–Ru(1)–N(8) 79.5(2); N(9)–Ru(1)–N(10) 78.89(18); N(2)–Ru(1)–N(8) 173.47(18); N(5)–Ru(1)–N(9) 178.59(19); N(7)–Ru(1)–N(10) 172.03(18).

Crystals of X-ray diffraction quality were obtained for both **1** and **2**. The structures of the cations are depicted in Figure 2 with both adopting distorted octahedral coordination geometries. Ru–N bond lengths are largely unremarkable and lie between 2.035 and 2.086 Å for the two cations; however, a slightly longer Ru–N bond for the pictz pyridine donor in **1** of 2.122 Å is observed, which may be due to conformation demands of the pictz ligand. Bond angles for mutually *trans* Ru–N bonds lie between 172.0 and 178.6°. The pictz and btzm ligands adopt 6-membered chelate rings with bite angles of 89.1(4) and 88.05(18)° in **1** and **2**, respectively, which are larger than the bite angle for the bpy ligands (∼79°) in both complexes due to their increased conformation flexibility. Both triazole-based ligands adopt boat-like conformation such that the methylene protons adopt distinct axial and equatorial positions. The angle between the plane defined by the ruthenium center and the two coordinated N-donor atoms and that defined by the methylene carbon atom and the two atoms of the heterocycles which it bridges is 62.8° in **1** while a corresponding angle of 63.7° is observed for **2**. Additionally, a significant angle of 63.9° is observed between the planes of the pyridine and triazole rings of the pictz ligand in **1**, while an angle of 46.1° is observed between the planes of the two triazole rings of the btzm ligand in **2**.

The asymmetry of the pictz ligand in **1** renders the two bpy ligands unique and makes all 16 bpy protons inequivalent by ^1^H NMR spectroscopy (Figure S5). The triazole ring proton gives rise to a singlet at δ 8.51 ppm in *d*_3_-acetonitrile, while the diastereotopic methylene protons appear as a geminal pair of roofed doublets at δ 5.65 and 5.84 ppm (^2^*J*_HH_ = 16.3 Hz). Conversely, the triazole ring protons of the btzm ligand in **2** give rise to a singlet resonance at δ 8.63 ppm with the methylene bridge protons of the ligand resulting in a singlet resonance at δ 6.81 ppm (Figure S7). A total of eight bpy proton resonances are also observed suggesting that the boat-like conformation of the btzm ligand undergoes fluxional inversion which is rapid on the NMR time scale.

### Electrochemical and Photophysical Properties

**Figure 3:**
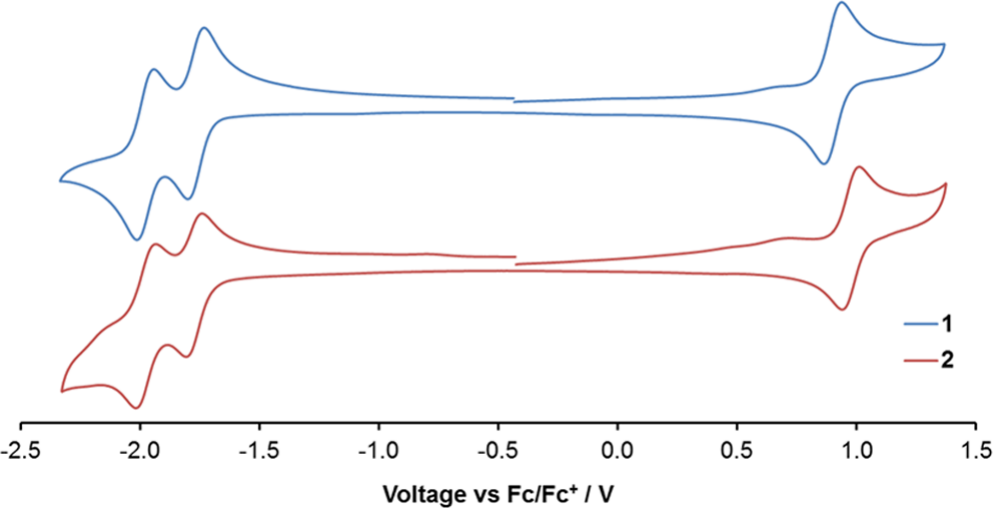
Cyclic voltammetry traces for **1** and **2** in acetonitrile with tetrabutylammonium hexafluorophosphate electrolyte and referenced relative to Fc/Fc^+^ (*E* = 0.0 V).

The electrochemical properties of **1** and **2** were investigated by cyclic voltammetry, and the data are presented in Table 1 and Figure 3. Both complexes show a reversible oxidation process corresponding to Ru(II)/Ru(III) couples and two reversible reduction processes, corresponding to one-electron reduction of each of the two bpy ligands. The oxidation process for **1** (+0.89 V vs Fc/Fc^+^) is identical to that of [Ru(bpy)_3_]^2+^ (+0.89 V) whereas this is shifted to more positive potential for **2** (+0.97 V). For comparison, for [Ru(bpy)_2_(btz)]^2+^ in which the two triazole donors coordinate through the N3 atoms of the ring rather than N2, the Ru(II)/Ru(III) process appears at +0.92 V. The first reduction potentials of **1** and **2** are almost identical and appear at −1.76 and −1.77 V, respectively, and are slightly shifted to more negative potential compared to that for [Ru(bpy)_3_]^2+^ (−1.73 V), which is suggestive of a slight destabilization of the ligand-based LUMO compared to the homoleptic complex.

**Table 1:** Summary of Spectroscopic and Electrochemical Data for Complexes **1** and **2**

complex	λ^abs^/nm (ε/dm^3^ mol^–1^ cm^–1^)^a^	λ^em^/nm^b^	*E*_red_/V^c^	*E*_ox_/V^c^
**1**	448 (8490), 411 (7720), 361 (7260), 328 (12,400), 287 (56,880)	566, 613, 667(sh)	–1.76 (74), −1.98 (64)	+0.89 (92)
**2**	425 (8260), 398 (8070) 354 (9160), 324(14,790), 283 (61,890)	551, 592, 645(sh)	–1.77 (74), −1.99 (84)	+0.97 (86)

a Acetonitrile solution at room temperature.

b 77 K, 4:1 EtOH/MeOH glass.

c Acetonitrile with N^n^Bu_4_PF_6_ electrolyte. Numbers in parentheses indicate anodic–cathodic peak separations (Δ*E*_a,c_).

**Figure 4:**
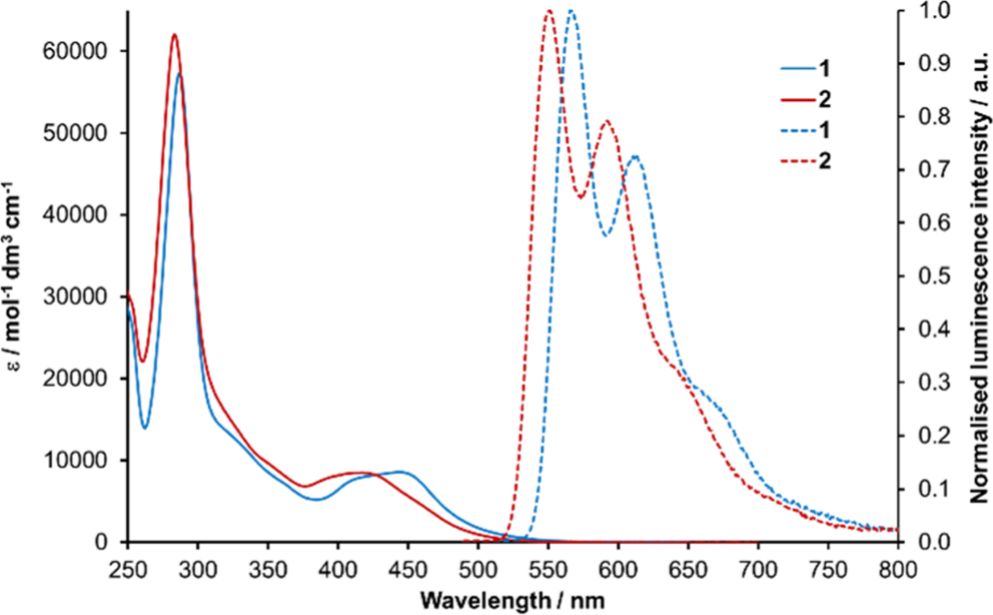
UV–visible absorption spectra in acetonitrile solutions at room temperature (solid traces) and emission spectra recorded at 77 K in ethanol/methanol (4:1) glass matrices (dashed traces) for **1** and **2**. λ^ex^ = 448 nm (**1**) and 415 nm (**2**).

Ultraviolet–visible (UV–vis) absorption spectra were recorded in acetonitrile solutions for **1** and **2**, and spectra are presented in Figure 4 with data summarized in Table 1. Both complexes exhibit intense bands around 280 nm assigned to bpy-based π → π* ^1^LC transitions with broad bands of lesser intensity between 400 and 450 nm assigned to bpy-dominated ^1^MLCT transitions. Also discernible are absorptions characterized by a tailing of the ^1^MLCT band beyond 500 nm which is ascribed to spin-forbidden direct ground state to ^3^MLCT state transitions. In line with the electrochemical data for **2**, which predicts a larger HOMO–LUMO separation, the ^1^MLCT maximum (425 nm) is blue-shifted when compared to **1** (448 nm). The spectra also appear to show evidence of additional absorption bands between 300 and 370 nm. For [Ru(bpy)_2_(btz)]^2+^ and [Ru(bpy)_2_(pytz)]^2+^, such absorption bands have been assigned as having ^1^MLCT character with charge transfer to the triazole-based ligand and are similarly positioned to ^1^MLCT absorption bands for [Ru(btz)_3_]^2+^ complexes for which there is little or no absorption at wavelengths longer than 400 nm.^36,53,54^ By comparison, we therefore assign these absorption features in this region for **1** and **2** to ^1^MLCT transitions involving the pictz and btzm ligands.

While emission is not discernible in room-temperature solutions, **1** and **2** are emissive at 77 K in solvent glass matrices (Figure 4 and Table 1). Luminescence spectra are characterized by structured bands with vibronic progressions. In agreement with the stabilization of the HOMO (as indicated by cyclic voltammetry) and the blue shift in the ^1^MLCT absorption band for **2** in comparison to **1**, the emission maximum for the high energy progression is also blue-shifted by 15 nm from 566 to 551 nm.

### Transient Absorption Spectroscopy

**Figure 5:**
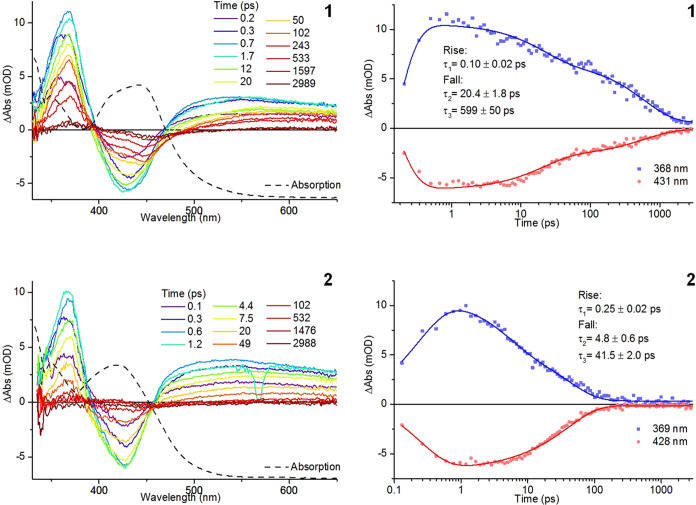
UV–visible transient absorption spectra (λ^ex^ = 285 nm) for **1** and **2** in acetonitrile with ground-state absorption spectrum overlaid (left) and temporal evolution of excited-state transient and ground-state bleach bands (right).

To gain an insight into the excited-state dynamic processes for **1** and **2**, we carried out a UV–visible transient absorption spectroscopic study on the two complexes. Both complexes exhibit TA spectra (Figure 5) that contain ground-state bleach features that are coincident with the position of ^1^MLCT bands between 400 and 450 nm. These are accompanied by excited-state absorption features at approximately 370 nm with a further broad absorption feature extending from ∼500 nm to longer wavelengths toward the red end of the spectrum and are assigned to the ^3^MLCT state in both cases. Short rise times for the appearance of transient and bleach features with τ_1_ = 0.10 ± 0.02 ps (**1**) and 0.25 ± 0.02 ps (**2**) were observed. Kinetic analysis of the transient decay and bleach recovery reveals two decay processes for both complexes. The shorter of these components, τ_2_, is assigned as a mixture of vibrational cooling, energy redistribution, and internal conversion processes, while the longer process, τ_3_, is assigned to the decay of the ^3^MLCT state to the ground state. For **1**, lifetimes for the two processes of τ_2_ = 20.4 ± 1.8 ps and τ_3_ = 599 ± 50 ps were determined. In the absence of room-temperature emission for these species decay of the ^3^MLCT state is assumed to occur with the involvement of ^3^MC states. Since the spectroscopic data indicate higher-energy ^1^MLCT and ^3^MLCT states for **2** compared to **1**, the deactivation of the ^3^MLCT state of **2** to populate ^3^MC states, which would be in closer proximity energetically, might be expected to be more rapid due to a lower activation barrier on the triplet potential energy surface. In agreement with this, the lifetimes fitted to the transient decay and bleach recovery indicate more rapid evolution for **2** compared to **1** with τ_2_ = 4.8 ± 0.6 ps and τ_3_ = 41.5 ± 2.0 ps.

Examination of difference spectra at 3 ns at the end of the available transient time window shows some evidence of low intensity but persistent negative and positive features. Most noticeable for **1**, these appear at approximately 450 and 375 nm, respectively, and may indicate partial photochemical consumption of the starting complexes during the measurement. The relative magnitude of these signals is inconsistent with what one might expect if the photochemical product formed is [Ru(bpy)_2_(NCMe)_2_]^2+^ given the differences in its visible absorption spectrum in these regions compared to that for **1**. However, formation of significant quantities of [Ru(bpy)_2_(NCMe)_2_]^2+^ under such flash photolysis conditions is unlikely. As 6-membered chelate complexes commonly undergo two-step photolysis through initial dechelation, it is therefore probable that these residual signals arise through the formation of one or more ligand-loss intermediate species (*vide infra*) which could account for this discrepancy.

### Photochemical Reactivity

**Figure 6:**
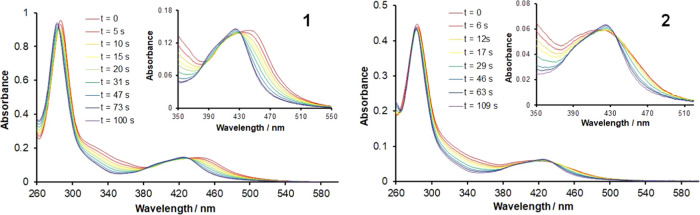
UV–visible absorption spectra recorded during the photolysis of **1** and **2** in acetonitrile solutions. Insets: magnification of ^1^MLCT regions in each case (λ^ex^ = 446 nm).

The photochemical reactivities of **1** and **2** were monitored by UV–visible spectroscopy in acetonitrile solutions. Representative spectra are provided in Figure 6 (emission spectrum of the LED excitation source is shown in Figure S9). In both cases, photolysis proceeds with noticeable bleaching of the spectrum in the region from 310 to 375 nm. For **1**, the bpy-centered ^1^MLCT absorptions are observed to diminish with the resultant maximum undergoing a blue shift. For **2**, the ^1^MLCT bands appear to sharpen and narrow and increase slightly in intensity. For both complexes, no change to spectra is observed beyond 3 min of irradiation time, at which point the spectra are near-identical and match that for [Ru(bpy)_2_(NCMe)_2_]^2+^ (Figure S10), indicating complete dissociation of the triazole-containing ligand. This is further supported by the bleaching of pictz- and btzm-associated ^1^MLCT bands between 300 and 370 nm.

A noticeable feature in the spectra of both complexes is that during the first 15 s, there are no isosbestic points indicating the formation of one or more intermediate species (see inset spectra in Figure 6). Beyond 15 s, the spectra do appear to exhibit clear isosbestic points, which appear at 381 and 432 nm in the ^1^MLCT region for **1** and at 399 and 434 nm for **2**. This is suggestive of a two-step process involving dechelation of the triazole-based ligand with subsequent ligand release. While ligand-loss intermediates are not, or at least are not always, observed for complexes undergoing photorelease of more rigid 5-membered chelate ligands,^27^ such intermediates are more commonly observed for complexes with 6-membered chelate ligands such as diamines^44^ and thioethers,^55^ presumably as a consequence of their greater conformational freedom.

To rule out potential thermal reactivity, samples of both complexes were dissolved in *d*_3_*-*acetonitrile and kept in the dark with periodic monitoring by ^1^H NMR spectroscopy over a period of 6 days and show very little if any change (Figure S11). Photochemical quantum yields for the overall ligand-loss processes for **1** and **2** of 0.079 and 0.091, respectively, were thus determined by the method reported by Slep and co-workers.^56^ These values represent a substantial enhancement in photochemical ligand release efficiency over previously reported ruthenium(II) triazole-based complexes containing 5-membered chelate rings (e.g., [Ru(bpy)_2_(btz)]^2+^ ϕ = 0.02, [Ru(bpy)_2_(pytz)]^2+^ ϕ = 0.003)^17^ and are comparable to that of [Ru(bpy)_2_(6,6′-Me_2_bpy)]^2+^.^27^

**Figure 7:**
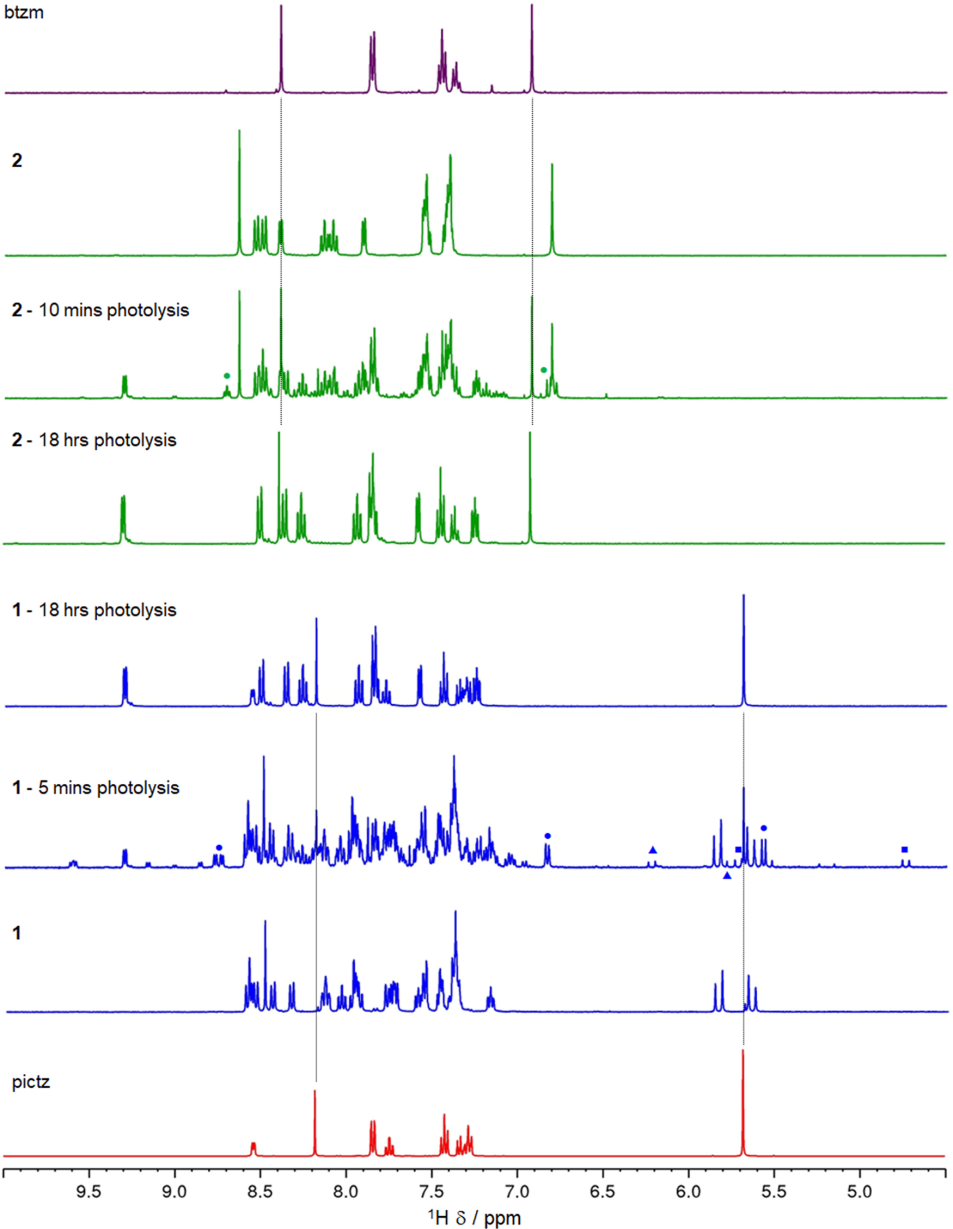
Stacked ^1^H NMR spectra (400 MHz) of the free ligands pictz and btzm, complexes **1** and **2**, and spectra recorded during and after prolonged photolysis of **1** and **2** (blue up-pointing triangle and blue square = **4-py** and **4-tz**^**(N2)**^, blue circle = **4-tz**^**(N3)**^, green circle = **4-tz**^**(N3)**^). All spectra were recorded in *d*_3_-acetonitrile. The light source used for photolysis was a 23 W domestic fluorescent light bulb (emission profile provided in the ESI).

The photolysis of **1** and **2** was also monitored by ^1^H NMR spectroscopy in *d*_3_-aceotnitrile with ex situ irradiation using the mercury emission lines of a 23 W fluorescent light bulb (Figure S9). Representative spectra are provided in Figure 7 for the two complexes. Photolysis proceeds at a markedly slower rate under these conditions compared to monitoring by UV–visible absorption spectroscopy when using the same light source. This is primarily due to the much higher concentrations required for NMR spectroscopy which takes the sample well beyond the optically dilute limit. In both cases, photolysis proceeds with the observed appearance and increasing intensities of signals for the free pictz and btzm ligands indicating their dissociation from the metal center. As the signals for the free ligand appear, the resonances for **1** and **2** are observed to diminish. A common set of signals are observed to grow during the photolysis of both **1** and **2**, which are assigned to the solvento complex [Ru(bpy)_2_(NCMe)_2_]^2+^ (**3**) and match spectral data for an authentic sample of **3** (Figure S12).^17^

**Figure 8:**
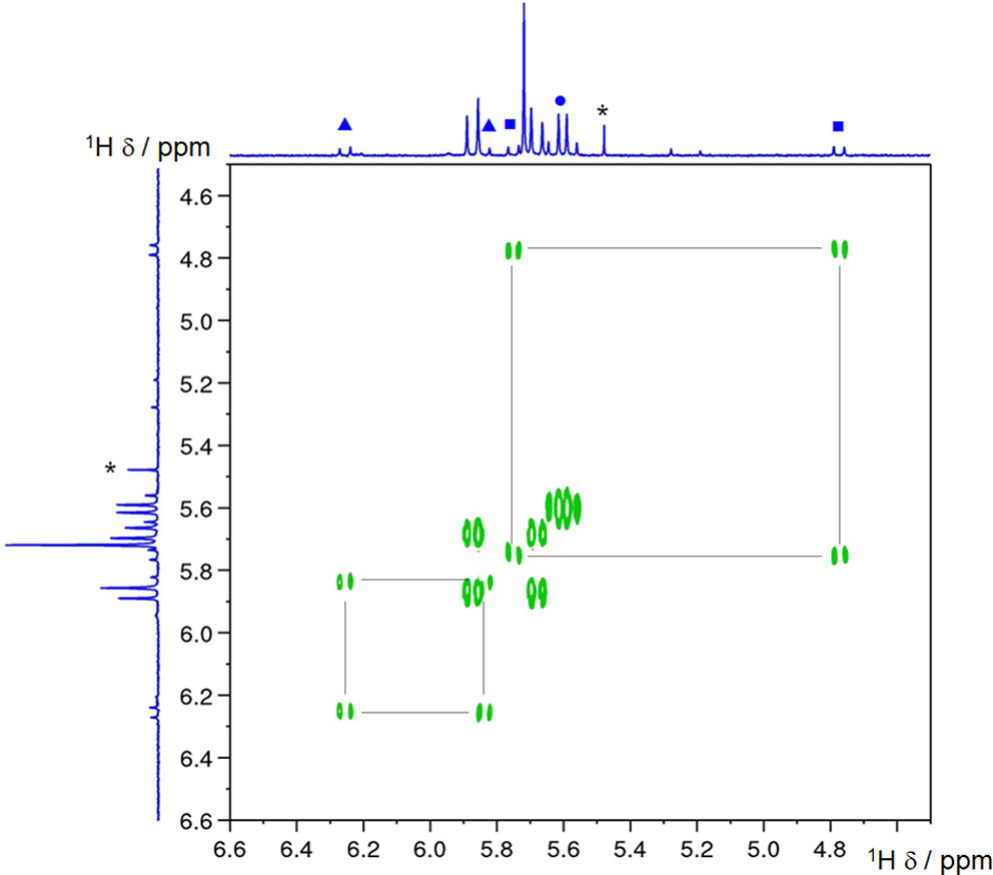
^1^H–^1^H COSY NMR spectrum recorded after 3 min of photolysis of **1** in *d*_3_-acetonitrile (* solvent impurity, blue up-pointing triangle and blue square = **4-py** and **4-tz**^**(N2)**^, blue circle = **4-tz**^**(N3)**^). The light source used for photolysis was a 23 W domestic fluorescent light bulb (emission profile provided in the ESI).

**Scheme 1:**
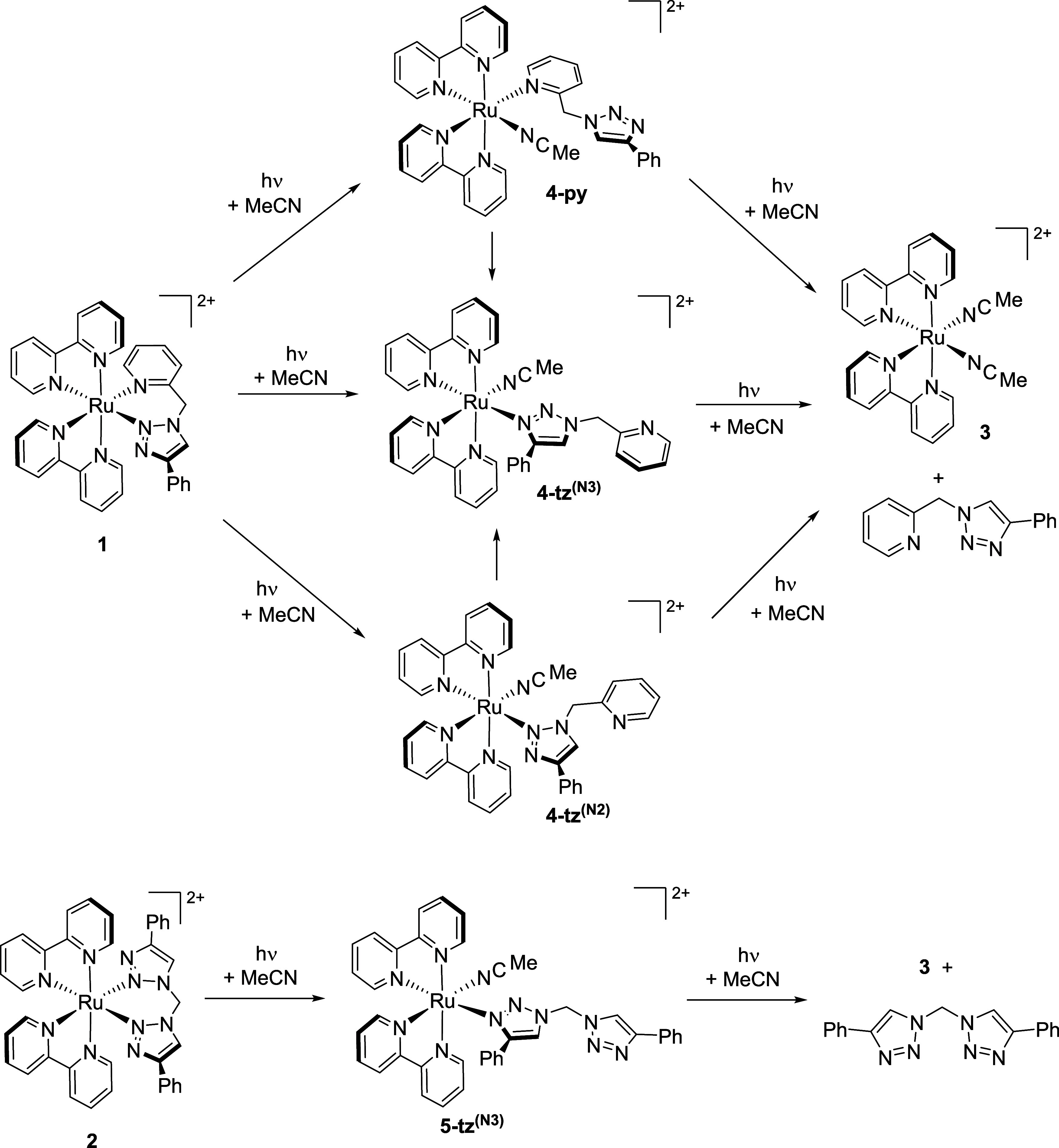
Proposed Photochemical Mechanisms for Ligand Ejection from **1** and **2**

For both **1** and **2**, additional resonances are observed during the early stages of photolysis which are assigned to ligand-loss intermediate species containing monodentate pictz and btzm ligands. During the first few minutes of photolysis of **1**, three ligand-loss intermediate species are identified which are characterized by the appearance of pairs of geminally coupled doublets assigned to the bridging methylene protons for monodentate pictz ligands (confirmed by ^1^H–^1^H COSY spectroscopy, Figure 8). Two of these are formed at lower concentrations and exhibit a very wide separation between constituent doublets of the pairs (δ 6.22 and 5.81 ppm (*J*_HH_ = 15.8 Hz) and δ 5.72 and 4.74 ppm (*J*_HH_ = 15.6 Hz) denoted by square and triangle symbols in Figures 7 and 8). The third of these intermediates exhibits resonances of greater intensity and a much-reduced separation between the doublets of the geminal pair (δ 5.60 and 5.55 ppm (*J*_HH_ = 15.1 Hz) denoted by circle symbols in Figures 7 and 8). The asymmetry of the pictz ligand presents the possibility of scission of either the Ru–N_triazole_ or Ru–N_pyridine_ bonds, which would result in two possible monodentate ligand-loss intermediates in which the pictz ligand is bound to the ruthenium(II) center by either the pyridine (**4-py**) or triazole (**4-tz**^**(N2)**^) donor, respectively (Scheme 1). From the NMR data, we propose that the two intermediates observed to form at a lower concentration during the photolysis of **1** can indeed be assigned to these intermediates. We rationalize the wide spacing of the doublets in these geminal pairs as arising due to the steric demands of the decoordinated and now pendant heterocycle which induces rotation about the methylene bridge to avoid unfavorable interactions with the other ligands coordinated to the metal. This would result in the methylene bridge becoming oriented such that the C–H bonds point more toward the core of the complex rather than away as in **1**, resulting in a far more significant difference in the magnetic environment for each proton of the diastereotopic methylene group. Unfortunately, data do not allow a definitive assignment of which doublet pair belongs to which dechelation intermediate; significant overlap of bpy and pyridine-based resonances for **1**, **3**, and the three intermediate complexes in the region δ 7.2 to 8.7 ppm makes NOE analysis extremely problematic. However, electrospray mass spectrometry analysis of the NMR sample at this point confirms the formation of solvento ligand-loss intermediates with detection of a monocation with *m*/*z* 839.16 consistent with an ion-pair of the form {[Ru(bpy)_2_(pictz)(NCCD_3_)]PF_6_}^+^ (Figure S13).

Thus far, this only accounts for the formation of two intermediate species; however, we note that the pictz ligand possesses a third potential donor N atom. We therefore assign the third, and the more closely spaced pair of geminal coupled doublet as arising from an intermediate containing a monodentate pictz ligand coordinated by the more basic triazole N3 atom (**4-tz**^**(N3)**^, Scheme 1). Given that monodentate triazole ligands favor coordination through the N3 atom of the ring such a rearrangement would be understandable. This assignment is further supported by the fact that this coordination mode would place the methylene bridge further from the influence of the chiral metal center, resulting in a smaller separation of the doublet resonances compared to the separation of the diastereotopic CH_2_ resonances of the other two ligand-loss intermediate species **4-py** and **4-tz**^**(N2)**^. Further, the appearance of the methylene proton resonances for **4-tz**^**(N3)**^ is accompanied by the observation of a new doublet resonance at δ 6.8 ppm tentatively assigned to the *o*-Ph protons of the phenyl substituent on the triazole ring. The two sets of signals are observed to grow in and decay together during the course of photolysis. Linkage isomerism to coordinate via the N3 atom would bring the phenyl substituent into closer promixity to the bpy ligands with which it may undergo π-stacking interactions and account for the observed shielding of this resonance due to ring current effects. Similar effects have been previously noted for the monodentate btz ligands in monodentate intermediates observed for [Ru(bpy)(btz)_2_]^2+^.^33,35^

Spectra recorded early in photolysis also exhibit weak singlet resonances at δ 5.25 and 5.16 ppm which disappear on complete photolysis. These could arise from *trans* isomers of the ligand-loss intermediates described above. Bis(bitriazolyl) complexes of ruthenium are known to form *trans* photoproducts,^34^ bis(bipyridyl) complexes have been shown to undergo *cis*/*trans* photoisomerism,^57^ and *trans* bis-solvent bis-bpy complexes have been crystallographically characterized.^58^ However, in the absence of further corroborative evidence, this remains a tentative assignment.

When a partially photolyzed sample exhibiting resonances for all three intermediates is left in the dark overnight and subsequently reexamined, resonances for **4-py**/**4-tz**^**(N2)**^ are observed to have disappeared while those assigned to **4-tz**^**(N3)**^ remain. While both **4-py** and **4-tz**^**(N2)**^ might be expected to undergo thermal loss of the solvent ligand and rechelation of the pictz ligand as observed in other triazole-based ligand-loss intermediates,^33,35^ this would not be possible for the linkage isomerized intermediate **4-tz**^**(N3)**^. The persistence of these resonances therefore further corroborates the assignment for the coordination mode of the pictz ligand in **4-tz**^**(N3)**^.

For **2**, a pair of roofed and narrowly separated geminal doublets for the methylene protons, partly obscured by the methylene resonance for **2** but confirmed by COSY data (Figure S14), are observed in spectra at δ 6.85 and 6.81 ppm (*J*_HH_ = 14.3 Hz) at early times during photolysis. While some other additional signals are discernible at this time, signals for a comparable ligand dechelation intermediate with a widely spaced of pair doublets as observed for **4-py**/**4-tz**^**(N2)**^ could not be identified. When a sample that has undergone partial photolysis is subsequently kept in the dark at room temperature and periodically monitored, signals for the intermediates are still observable. Due to the similar appearance of their methylene resonances to those of **4-tz**^**(N3)**^ and their persistence in the spectra in the dark, these resonances are assigned as a ligand-loss intermediate containing a monodentate btzm ligand coordinated via the N3 atom of one of the triazole rings (**5-tz**^**(N3)**^, Scheme 1). After prolonged photolysis signals for these intermediate species disappear and only resonances for [Ru(bpy)_2_(NCMe)_2_]^2+^ and free btzm are observed. Electrospray mass spectrometry confirms the formation of solvento ligand-loss intermediates with the detection of a monocation with *m*/*z* 905.18 consistent with an ion-pair {[Ru(bpy)_2_(btzm)(NCCD_3_)]PF_6_}^+^ (Figure S13).

**Scheme 2:**
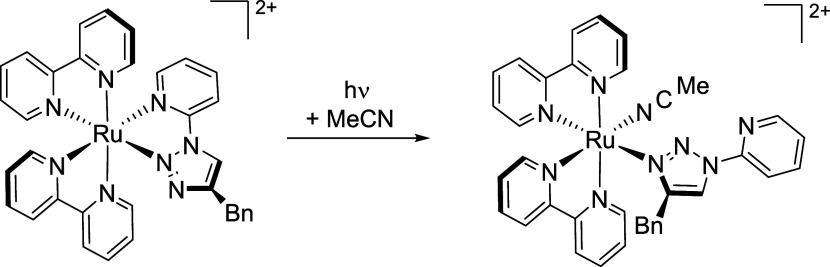
Reinterpretation of Photochemical Reactivity of [Ru(bpy)_2_(pytz^inv^)]^2+^ in Acetonitrile^43^

Some of us have previously reported the observation of a ligand-loss intermediate during the photolysis of the complex [Ru(bpy)_2_(pytz^inv^)]^2+^ (pytz^inv^ = 4-benzyl-1-(pyrid-2-yl)-1,2,3-triazole, Scheme 2) in which the 5-membered chelate pytz^inv^ ligand (which lacks the methylene spacer present in pictz) is similarly forced to coordinate through the less basic triazole N2 atom in order to chelate.^43^ This species appeared to show similar longevity to that observed for **4-tz**^**(N3)**^ and **5-tz**^**(N3)**^. This was originally proposed to arise through simple scission off the of Ru–N(py) bond yielding a species in which the pytz^inv^ ligand is coordinated via the N2 atom of the triazole ring. However, based on the data in this study, we therefore reason that the observed intermediate in this previous study may in fact be a comparable rearrangement product in which the pytz^inv^ ligand is coordinated through its triazole N3 atom (Scheme 2).

To probe the fate of **4-py** and **4-tz**^**(N2)**^, a partially photolyzed sample of **1** for which the three intermediates are observed to be present was kept in the dark in the bore of the spectrometer overnight at 298 K and ^1^H NMR spectra recorded every 20 min. As discussed above, resonances for these species are observed to slowly reduce in intensity and ultimately disappear while those attributed to **4-tz**^**(N3)**^ remain (Figure S15). A possible explanation for this observation would be rechelation of the pictz ligand upon dissociation of the solvent ligand and reformation of **1**. However, when the relative integrations of **1**, **4-py/4-tz**^**(N2)**^, **4-tz**^**(N3)**^, and free pictz are extracted (referenced to solvent, Figure S16) and examined over the duration of this experiment, the concentration of **1** is not observed to increase as concentrations of **4-py** and **4-tz**^**(N2)**^ diminish indicating that rechelation does not occur. On the other hand, the data reveal that the concentrations of **4-tz**^**(N3)**^ and free pictz both increase indicating that both linkage isomerism and pictz dissociation are occurring. Based on the overall changes, we estimate that approximately 25% of the combined intermediates **4-py** and **4-tz**^**(N2)**^ undergo ligand dissociation while 75% undergo linkage isomerism to form **4-tz**^**(N3)**^.

The data described above suggests that the monodentate pictz ligand is somewhat thermally labile and that at least some **4-py** (in addition to **4-tz**^**(N2)**^) may convert to **4-tz**^**(N3)**^. We therefore propose that pictz undergoes slow thermal dissociation in both **4-py** and **4-tz**^**(N2)**^ and that conversion to **4-tz**^**(N3)**^ occurs through recoordination via the triazole N3 atom while the ligand remains within the solvent cage in a process that is competitive with formal loss of pictz and formation of **3**. However, this process is too slow to account for the rapid formation of **4-tz**^**(N3)**^ under photochemical conditions, and its observation in the very first spectrum recorded on initiating photolysis thereby indicates that it is primarily formed through a light-driven process under these conditions.

**Figure 9:**
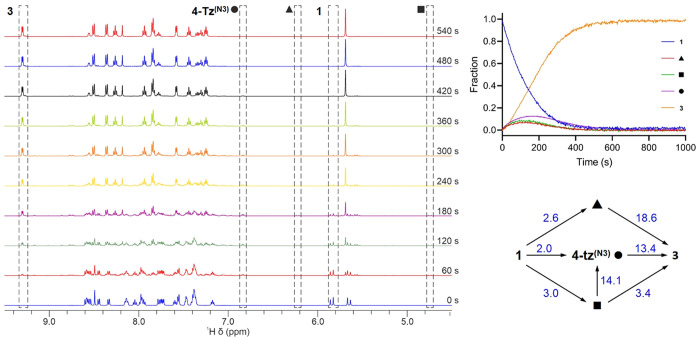
Left: ^1^H NMR spectra recorded during NMRtorch illumination (λ^ex^ = 459 nm) of complex **1**. The sample was irradiated with a 2 s pulse prior to each spectral acquisition. The selected spectra are taken after successive 60 s cumulative irradiation periods with signals used in kinetic analyses highlighted (starting material **1**, photoproduct [Ru(bpy)_2_(NCMe)_2_]^2+^ (**3**), and intermediates **4-tz**^**(N3)**^ (labeled ●) and **4-py**/**4-tz**^**(N2)**^ (labeled ▲/■)). Starting material **1** integral was corrected to remove the contribution from an overlapping signal from the intermediate (▲). Right: Evolution with time of the mole fractions for complex **1**, photoproduct **3**, and intermediates **4-tz**^**(N2)**^ and **4-py** (■/▲) and **4-tz**^**(N3)**^ (●) determined by ^1^H NMR spectroscopy with in situ photolysis (λ^ex^ = 459 nm) with kinetic fitting. Kinetic model scheme used for fitting and calculated rate constants (×10^–3^% s^–1^) from fitting in Dynafit 4 software.

Real-time in situ photolysis ^1^H NMR spectra were recorded using the recently reported NMRtorch apparatus developed by Golovanov,^59^ the first time this method has been applied to photochemically reactive metal complexes. We were interested in determining whether **4-tz**^**(N3)**^ undergoes delayed formation after an induction period as a secondary photoproduct from **4-tz**^**(N2)**^ and/or **4-py**, or whether all three intermediates form divergently and directly from **1**. The NMR spectra and extracted mole fractions of **1**, **3**, and intermediates assigned as **4-py**, **4-tz**^**(N2)**^, and **4-tz**^**(N3)**^ as a function of time are shown in Figure 9 and show that all three intermediate species are observed to grow in together with no delay on the appearance of **4-tz**^**(N3)**^. The intermediates **4-tz**^**(N2)**^ and **4-py** are observed to have largely disappeared by 400 s, whereas **4-tz**^**(N3)**^ continues to decay and has mostly disappeared after 600 s with photolysis fully complete after approximately 700 s.

The mole fraction data for the photolysis of **1** was fitted using DynaFit software to various models involving direct-only formation of **4-tz**^**(N3)**^, indirect-only formation of **4-tz**^**(N3)**^ from either **4-tz**^**(N2)**^ or **4-py**, and models involving both direct and indirect formation of **4-tz**^**(N3)**^ (see Supporting Information, Figures S17–S20). While no clearly definitive model was identified from this fitting, models which best fit the experimental data involve both direct formation of **4-tz**^**(N3)**^ from **1** in addition to indirect formation from one of the other two intermediates. Indeed, a model featuring only direct formation of the three intermediates from **1** and direct exclusive conversion of each intermediate to **3** leads to a poorer fit to the experimental data (Figure S18). The best model from those surveyed suggests that the intermediate giving rise to the most shielded CH_2_ resonance (■ in Figure 9) is photochemically converted into **4-tz**^**(N3)**^ at a rate almost 5 times that for its conversion to **3** through loss of the pictz ligand (Figure 9, right). Based on this, one might tentatively assign this intermediate to be **4-tz**^**(N2)**^ since with the triazole ring already coordinated this would be consistent with a relatively efficient linkage isomerism to **4-tz**^**(N3)**^. For **2**, the experimental data can be adequately fit to a two-step model involving conversion of **2** to **3** via an intermediate with rate constants of 0.026 and 0.053% s^–1^ for the first and second steps, respectively (Figure S20).

### Mechanistic Implications

We now turn to potential mechanistic implications of the results outlined above. Photochromic rearrangement reactions as well as photochemical ligand loss are well known to proceed via population of ^3^MC states which are thermally populated from ^3^MLCT states^11,19^ (however, evidence has also been provided for photochemical rearrangement and ligand release from the ^3^MLCT state in some complexes^60,61^). Photochromic rearrangement reactions of metal complexes may occur through traversing the lowest triplet potential energy surface of the complex, transiting through multiple minima exhibiting ^3^MLCT and ^3^MC character before crossing to the ground-state surface of the photoproduct.^62,63^ In recent work, Dixon and co-workers have demonstrated that it is energetically feasible that ligand dechelation, solvent capture, and bidentate ligand release may all occur in the triplet manifold as part of a single photochemical reaction without the involvement of ground-state intermediate species.^20,64^

It is therefore possible that **1** undergoes divergent photochemical reactivity to directly form the three observed intermediate species **4-tz**^**(N3)**^, **4-tz**^**(N2)**^, and **4-py**. Formation of **4-tz**^**(N2)**^ and **4-py** may occur via ^3^MC state population with induced pictz ligand dechelation after Ru–N bond elongation followed by solvent capture. For **4-tz**^**(N3)**^, solvent capture and formal pictz ligand release may occur with subsequent recoordination of pictz through the triazole N3 atom before escape from the solvent cage yielding the observed ligand-loss intermediate. This process could be competitive with escape of pictz from the solvent cage and account for the very early observation of free pictz in NMR spectra. Alternatively, solvent capture followed by intramolecular rearrangement via population of a series of states of ^3^MLCT and/or ^3^MC character may occur without formal release of the pictz as has been implicated in S- to O-donor photochromic rearrangement reactions.^62^ While it may be possible for **3** and free pictz to partially be formed in a one-step photochemical process, the final photoproducts are evidently also formed in a two-step process with photochemical release of pictz from the intermediates **4-tz**^**(N3)**^, **4-tz**^**(N2)**^, and **4-py**. Similar processes for **2** would account for the formation of the ligand-loss intermediate **5-tz**^**(N3)**^ and formation of **3** and free btzm.

While computational studies could offer additional insight into these processes and their relative feasibilities, this would present a far from trivial task. The work nevertheless highlights highly intriguing and novel photochemical reactivity for ruthenium(II) triazole-based complexes.

## Conclusions

Complexes bearing 1,2,3-triazole-based ligands which form 6-membered chelate rings, and in which chelation requires less favorable coordination of the triazole ring by its N2 rather than N3 atom, undergo rapid photochemical release of the triazole-based ligand. Further, this occurs with the observation of ligand-loss intermediates, most interestingly including intermediates in which the monodentate triazole-containing ligand has undergone linkage isomerism and coordinates by the more basic triazole N3 atom. While intermediates derived from simple dechelation of the triazole-based ligand are shown to slowly convert thermally to the linkage isomer in the dark, this linkage isomer is observed to be present in NMR spectra from the outset under photolysis. It is therefore likely that it also forms directly from the starting complex by a single photochemical process under irradiation rather than solely through a sequential mechanism involving other dechelated intermediates. The observations in this work provide an interesting example of chelate ligand release photochemistry, but may also provide possible avenues for exploitation in areas of photoactivated chemotherapy where a pharmacologically active triazole-based ligand may be released, and in novel light-activated molecular machines, switches, and ratchets.

## Experimental Section

### General Methods

Unless otherwise stated, all reagents were purchased from commercial sources and used without further purification. The solvents used were of laboratory grade, with ether referring to diethyl ether. 0.1 M Ammonium hydroxide/ethylenediaminetetraacetic acid (NH_4_OH/EDTA) solution for washing out copper salts from alkyne/azide coupling reactions was made up by mixing 30 g of EDTA with 900 mL of water and 100 mL of NH_4_OH.

^1^H and ^13^C NMR spectra were recorded on Bruker 400, 500, and 600 Avance NMR spectrometers at 298 K. Chemical shifts (δ) are reported in parts per million (ppm) and referenced to residual solvent peaks (CDCl_3_: ^1^H δ 7.26 ppm, ^13^C δ 77.16 ppm; CD_3_CN: ^1^H δ 1.94 ppm, ^13^C δ 118.26, 1.32 ppm). Coupling constants (*J*) are reported in hertz (Hz). Standard abbreviations indicating multiplicity were used as follows: m = multiplet, q = quartet, t = triplet, dt = double of triplet, d = doublet, dd = double doublet, s = singlet. IR spectra were recorded on a Bruker α FT-IR spectrometer with an attached α-P measurement module. Electrospray mass spectra (HRMS-ESI) were collected on a Bruker microTOF-Q or Agilent 6530 QTOF spectrometer. UV–visible absorption spectra were recorded on an Agilent Cary-60 spectrophotometer utilizing quartz cuvettes of 10 mm path length. Photoluminescence spectra were recorded on a Horiba Fluoromax-4 spectrophotometer at 77 K in an EtOH/MeOH glassing mixture.

### Electrochemistry

Cyclic voltammograms were measured using a PalmSens EmStat3 potentiostat with PSTrace electrochemical software. Analyte solutions with a typical concentration of 1.5 mmol dm^–3^ were prepared using dry MeCN, freshly distilled from CaH_2_. The supporting electrolyte was NBu_4_PF_6_, being recrystallized from EtOH and oven-dried prior to use with a typical solution concentration of 0.2 mol dm^–3^. The working electrode was a glassy carbon disk, Pt wire was used as a counter electrode, and the reference electrode was Ag/AgCl, being chemically isolated from the analyte solution by an electrolyte-containing bridge tube tipped with a porous frit. All potentials are quoted relative to the Fc^+^/Fc couple as an internal reference.

### Photochemistry

Photolysis experiments were carried out by irradiating the appropriate solutions contained within either NMR tubes or 10 mm path length quartz cuvettes with a blue LED (17 mW) or compact 23 W fluorescent light bulb (Hg). Samples were maintained at room temperature (25 °C) throughout the measurements with the aid of a Peltier temperature-controlled cuvette holder or an electronic fan (NMR samples). The determination of photochemical quantum yields was performed for MeCN solutions of known concentration (2.5 mL volume, 10 mm path length cuvette) under irradiation with a blue LED excitation source (17 mW, λ = 446 nm), the photon flux density of which was determined to be 1.94 × 10^–5^ einstein s^–1^ dm^–3^ through use of a K_3_Fe(C_2_O_4_)_3_·3H_2_O chemical actinometer. Calculations were performed using GNU Octave software (version 6.2.0), freely available at https://www.gnu.org/software/octave/, using the method of Slep and co-workers.^56^

For NMR experiments with in situ sample illumination, the NMRtorch^59^ was used with a peak maximum of 459 nm and LED supply power set to 1 W. Light pulses of 2 s duration were applied immediately before acquiring each NMR spectrum, to ensure that the photolysis reaction does not complete too fast to be observable.

### Transient Absorption Spectroscopy

Spectra were recorded using a broadband ultrafast pump–probe transient absorption spectrometer “Helios” (Ultrafast Systems LLC), collecting data over a 3 ns time window with a time resolution of approximately 250 fs. A Ti/sapphire amplifier system (Newport Spectra Physics, Solstice Ace) producing 800 nm pulses at 1 kHz with 100 fs pulse duration was used to generate the probe beam and to also pump a TOPAS Prime OPA with associated NIR-UV–vis unit to generate the excitation beam. The probe beam consisted of a white light continuum generated in a CaF_2_ crystal. Absorbance changes were monitored between 330 and 650 nm. Samples were excited with 0.5 μJ pulses at 285 nm, contained within a 0.2 cm path length quartz cuvette that was magnetically stirred during the measurements. Before data analysis, pre-excitation data was subtracted, and spectral chirp was corrected for.

#### Synthesis of ((4-Phenyl-1*H*-1,2,3-triazol-1-yl)methyl)pyridine (pictz)

2-(Bromomethyl)pyridine hydrobromide (0.25 g, 0.974 mmol, 1.0 equiv), sodium carbonate (0.62 g, 5.887 mmol, 6.0 equiv), sodium azide (0.084 g, 1.292 mmol, 1.3 equiv), ascorbic acid (0.14 g, 0.801 mmol, 0.8 equiv), copper(II) sulfate pentahydrate (0.10 g, 0.400 mmol, 0.4 equiv), and phenylacetylene (0.11 g, 1.087 mmol, 1.1 equiv) were combined in DMF/water (4:1, 15 mL). The resulting suspension was stirred at room temperature for 18 h, during which time the crude product precipitated as an off-white solid. The crude product was redissolved in CHCl_3_/^*i*^PrOH (3:1, 200 mL), and an aqueous solution of 0.1 M NH_4_OH/EDTA (200 mL) was added. The mixture was stirred vigorously at room temperature for 48 h. The organic phase was washed with water (100 mL) and saturated aqueous NaCl (100 mL), and dried over Na_2_SO_4_. The solvent was removed *in vacuo* to give the product as an off-white solid (0.134 g, 57%). ^1^H NMR (400 MHz, CDCl_3_): 5.67 (s, 2H, *CH*_2_), 7.17–7.26 (m, 2H, *Py*), 7.30 (tt, *J* = 1.2, 7.4 Hz, 1H, *Ph*), 7.38 (t, *J* = 7.6 Hz, 2H, *Ph*), 7.66 (td, *J* = 1.7, 7.8 Hz, 1H, *Py*), 7.81 (dd, *J* = 1.3, 7.9 Hz, 2H, *Ph*), 7.92 (s, 1H, *Tz*), 8.59 (d, *J* = 4.4 Hz, 1H, *py*). ^13^C NMR (101 MHz, CDCl_3_): 55.78, 120.30, 122.49, 123.50, 125.78, 128.23, 128.87, 130.61, 137.43, 148.27, 149.85, 154.58. HRMS-ESI (CH_2_Cl_2_/CH_3_OH) *m*/*z* = 237.1108 [M + H]^+^ (calcd for C_14_H_13_N_4_ 237.1135), *m*/*z* = 259.0928 [M + Na]^+^ (calcd for C_14_H_12_N_4_Na 259.0954). Spectroscopic data were consistent with that previously reported for this ligand.^51^

#### Synthesis of Bis(4-phenyl-1*H*-1,2,3-triazol-1-yl)methane (btzm)

Sodium carbonate (0.141 g, 1.330 mmol, 1.0 equiv) and sodium azide (0.077 g, 1.184 mmol. 0.9 equiv) were combined in DMF (8 mL) and DCM (0.5 mL, 7.830 mmol, 6.0 equiv) and irradiated in a microwave reactor for 20 min at 60 °C, then 20 min at 110 °C (200 W) to generate diazidomethane (**WARNING!** Low-molecular-weight azides, and diazides, are potentially explosive and should never be isolated, but only generated in situ!). Sodium l-ascorbate (0.094 g, 0.474 mmol, 0.4 equiv), copper(II) sulfate pentahydrate (0.060 g, 0.240 mmol, 0.2 equiv), phenylacetylene (0.133 g, 1.302 mmol, 1.0 equiv), and water (2 mL) were then added to the yellow solution, and the resulting suspension was stirred at room temperature for 18 h, during which time the crude product precipitated as an off-white solid. The crude product was redissolved in CHCl_3_/^*i*^PrOH (3:1, 200 mL), and an aqueous solution of 0.1 M NH_4_OH/EDTA (200 mL) was added. The mixture was stirred vigorously at room temperature for 1 h. The organic phase was separated, washed with water (100 mL) followed by saturated aqueous NaCl (100 mL), and dried over Na_2_SO_4_. The solvent was removed *in vacuo* to give the product as an off-white solid (0.103 g, 52%). ^1^H NMR (400 MHz, CDCl_3_): 6.89 (s, 2H, *CH*_2_), 7.35 (t, *J* = 7.2 Hz, 2H, *Ph*), 7.42 (t, *J* = 7.4 Hz, 4H, *Ph*), 7.80 (dd, *J* = 1.4, 7.8 Hz, 4H, *Ph*), 8.05 (s, 2H, *Tz*). ^13^C NMR (151 MHz, CDCl_3_): 61.07, 119.73, 126.04, 128.97, 129.11, 129.58, 149.54. IR: ν (cm^–1^) 3076, 1476, 1459, 1229, 1079, 754, 687, 517; HRMS-ESI: (CH_2_Cl_2_/CH_3_OH): *m*/*z* = 325.1156 [M + Na]^+^ (calcd for C_17_H_14_N_6_Na 325.1172), *m*/*z* = 627.2378 [M_2_ + Na]^+^ (calcd for C_34_H_28_N_12_Na 627.2452); Anal. Calcd for C_17_H_14_N_6_: C, 67.54; H, 4.67; N, 27.80. Found: C, 67.36; H, 4.75; N, 27.70.

#### Synthesis of [Ru(bipy)_2_(pictz)](PF_6_)_2_ (**1**)

[Ru(bpy)_2_Cl_2_] (0.081 g, 0.167 mmol, 1.00 equiv) and pictz (0.042 g, 0.176 mmol, 1.05 equiv) were combined in ethanol (10 mL). The suspension was deaerated with argon and then irradiated in a microwave reactor for 2 h (125 °C, 200 W). The orange solution was cooled to room temperature, and saturated aqueous NH_4_PF_6_ (30 mL) was added dropwise. The resulting precipitate was collected by vacuum filtration and washed with cold water (10 mL), cold ethanol (10 mL), and ether (10 mL) to give the orange product (0.110 g, 70%). ^1^H NMR (600 MHz, CD_3_CN): 5.65 (d, *J* = 16.3 Hz, 1H), 5.84 (d, *J* = 16.3 Hz, 1H), 7.19 (ddd, *J* = 1.5, 6.1, 7.5 Hz, 1H), 7.34–7.43 (m, 5H), 7.46–7.50 (m, 2H), 7.54–7.58 (m, 2H), 7.61 (ddd, *J* = 1.3, 5.7, 7.7 Hz, 1H), 7.74 (d, *J* = 5.7 Hz, 1H), 7.76 (d, *J* = 5.6 Hz, 1H), 7.79 (d, *J* = 7.7 Hz, 1H), 7.95 (td, *J* = 1.5, 7.7 Hz, 1H), 7.96–8.00 (m, 2H), 8.50 (td, *J* = 1.5, 7.9 Hz, 1H), 8.15 (tdd, *J* = 1.5, 3.2, 7.8 Hz, 2H), 8.34 (d, *J* = 8.2, 1H), 8.46 (d, *J* = 8.2, 1H), 8.51 (s, 1H), 8.55 (d, *J* = 8.2 Hz, 1H), 8.59 (d, *J* = 5.4 Hz, 1H), 8.60 (d, *J* = 7.9 Hz, 1H). ^13^C NMR (151 MHz, CD_3_CN): 54.88, 124.01, 125.13, 125.61, 125.63, 126.27, 127.10, 127.41, 127.51, 128.24, 128.28, 128.53, 129.09, 129.70, 130.00, 130.09, 138.76, 138.78, 138.94, 139.15, 139.70, 150.18, 152.75, 153.80, 154.00, 154.13, 154.51, 155.66, 158.20, 158.61, 158.78, 159.29. IR: ν (cm^–1^) 1467, 1442, 828, 756, 553; HRMS-ESI *m*/*z* calcd for [C_34_H_28_N_8_RuPF_6_]^+^: 795.1122, found: 795.1135 (M^+^), *m*/*z* calcd for [C_34_H_28_N_8_Ru]^2+^: 325.0734, found: 325.0750 (M^2+^). Anal. Calcd for C_34_H_28_N_8_RuP_2_F_12_ (%): C 43.46, H 3.00, N 11.93, found (%): C 43.37, H 3.03, N 11.93.

#### Synthesis of [Ru(bipy)_2_(btzm)](PF_6_)_2_ (**2**)

[Ru(bpy)_2_Cl_2_] (0.024 g, 0.050 mmol, 1.00 equiv) and btzm (0.015 g, 0.050 mmol, 1.00 equiv) were combined in 7:3 (*v*/*v*) ethanol/water (5 mL). The suspension was deaerated with argon and then irradiated in a microwave reactor for 16 h (120 °C, 200 W). The orange solution was cooled to room temperature, and saturated aqueous NH_4_PF_6_ (30 mL) was added dropwise. The resulting precipitate was collected by vacuum filtration and washed with cold water (10 mL) and ether (10 mL) to give the orange product (0.037 g, 74%). ^1^H NMR (400 MHz, CD_3_CN): 6.81 (s, 2H), 7.31–7.47 (m, 8H), 7.48–7.61 (m, 6H), 7.90 (d, *J* = 5.4 Hz, 2H), 8.08 (td, *J* = 1.3, 7.9 Hz, 2H), 8.13 (td, *J* = 1.3, 7.9 Hz, 2H), 8.39 (d, *J* = 5.4 Hz, 2H), 8.48 (d, *J* = 8.2 Hz, 2H), 8.53 (d, *J* = 8.5 Hz, 2H), 8.63 (s, 2H). ^13^C NMR (151 MHz, CD_3_CN): 62.02, 124.30, 125.00, 126.38, 127.51, 128.04, 128.39, 129.04, 130.06, 130.40, 139.10, 139.31, 150.05, 153.65, 154.00, 158.95, 159.11. IR: ν (cm^–1^) 1468, 1444, 835, 761, 555; HRMS (ES) *m*/*z* calcd for [C_37_H_30_N_10_RuPF_6_]^+^: 861.1334, found: 861.1340 (M^+^), *m*/*z* calcd for [C_37_H_30_N_10_Ru]^2+^: 358.0843, found: 358.0853 (M^2+^). Anal. Calcd for C_37_H_30_N_10_RuP_2_F_12_ (%): C 44.19, H 3.01, N 13.93, found (%): C 43.46, H 2.32, N 13.68.

### Crystallographic Methods

Crystals of **1** and **2** were grown via vapor diffusion of diethyl ether into acetonitrile solutions of the complexes. Both structures were collected on a Bruker Kappa Apex II area detector diffractometer using monochromated Mo Kα radiation (0.71073 Å) at low temperature (100 K). SADABS^65^ was used for absorption correction. The structures were solved by direct methods using either SIR-97^66^ or X-Seed^67^ and refined against F^2^ using anisotropic thermal displacement parameters for all nonhydrogen atoms using SHELXL-97^68^ or SHELXL-2014 software. Hydrogen atoms were placed in calculated positions and refined using a riding model. Due to the extent of disordered solvent in the crystal lattice of **2**, the SQUEEZE routine within PLATON was implemented, the details of which are contained within the relevant CIF.

Crystallographic data for **1**: C_34_H_28_F_12_N_8_P_2_Ru, *M*_r_ 939.65, orthorhombic, *Pna*2_1_, *a* = 12.8984(5) Å, *b* = 18.5088(5) Å, *c* = 15.2001(5) Å, α = 90°, β = 90°, γ = 90°, V = 3628.8(2) Å^3^, *Z* = 4, ρc = 1.720 Mg/m^3^; 28103 reflections collected, 7838 independent reflections (*R*_int_ = 7.36%), which were used in all calculations; *R*1 = 0.0539, w*R*2 = 0.1314 for I > 2σ(I) and *R*1 = 0.0847, w*R*2 = 0.1583 for all unique reflections; maximum and minimum residual electron densities 0.972 and −1.213 e Å^–3^. CCDC: 2323924.

Crystallographic data for **2**: C_37_H_30_F_12_N_10_P_2_Ru, *M*_r_ 1005.72, monoclinic, *C*2/*c*, *a* = 24.5648(4) Å, *b* = 14.9040(3) Å, *c* = 24.3024(4) Å, α = 90°, β = 94.337(2)°, γ = 90°, V = 8872.0(3) Å^3^, Z = 8, ρc = 1.506 Mg/m^3^; 46887 reflections collected, 8117 independent reflections (*R*_int_ = 7.37%), which were used in all calculations; *R*1 = 0.0772, w*R*2 = 0.2186 for I > 2σ(I) and *R*1 = 0.0825, wR2 = 0.2253 for all unique reflections; maximum and minimum residual electron densities 2.820 and −2.004 e Å^–3^. CCDC: 2323923.

## Supporting Information Available

^1^H and ^13^C NMR spectra for ligands and complexes and additional data for photochemical reactivity of complexes (mass spectrometry, UV–visible, and NMR spectroscopy, thermal dark stability of starting complexes and intermediates) (PDF). Additional data available at https://doi.org/10.17632/p4zwk5yg9z.1.
