# Supplementary material for: Photochemistry of Ru(II) Triazole Complexes with 6-Membered Chelate Ligands: Detection and Reactivity of Ligand-Loss Intermediates
Source: Inorg Chem. 2024 May 3;63(20):9084–97. doi: 10.1021/acs.inorgchem.4c00251 (PMC11110011; doi:10.1021/acs.inorgchem.4c00251)
Supplement: Supplementary file 1 — ic4c00251_si_001.pdf [file ic4c00251_si_001.pdf]

Supporting Information for

Photochemistry of Ru(II) triazole complexes with 6-membered chelate ligands: detection and reactivity of ligand-loss intermediates

Katie Eastham,[a] Aaron D. W. Kennedy,[b] Synøve Scottwell,[b] Jack E. Bramham,[d] Samantha Hardman,[c] Alexander P. Golovanov,[d] Paul A. Scattergood, [a]* James D. Crowley[b]* and Paul I. P. Elliott[a]*

* corresponding authors: p.i.elliott@hud.ac.uk; jcrowley@chemistry.otago.ac.nz; p.scattergood@hud.ac.uk

**NMR spectra for ligands and complexes**	**S2**
**Photochemical studies**	**S6**
**Stability studies of intermediate photoproducts**	**S11**
***In-situ* real-time kinetic modelling of photochemical reactivity by NMR**	**S12**

NMR spectra for ligands and complexes.

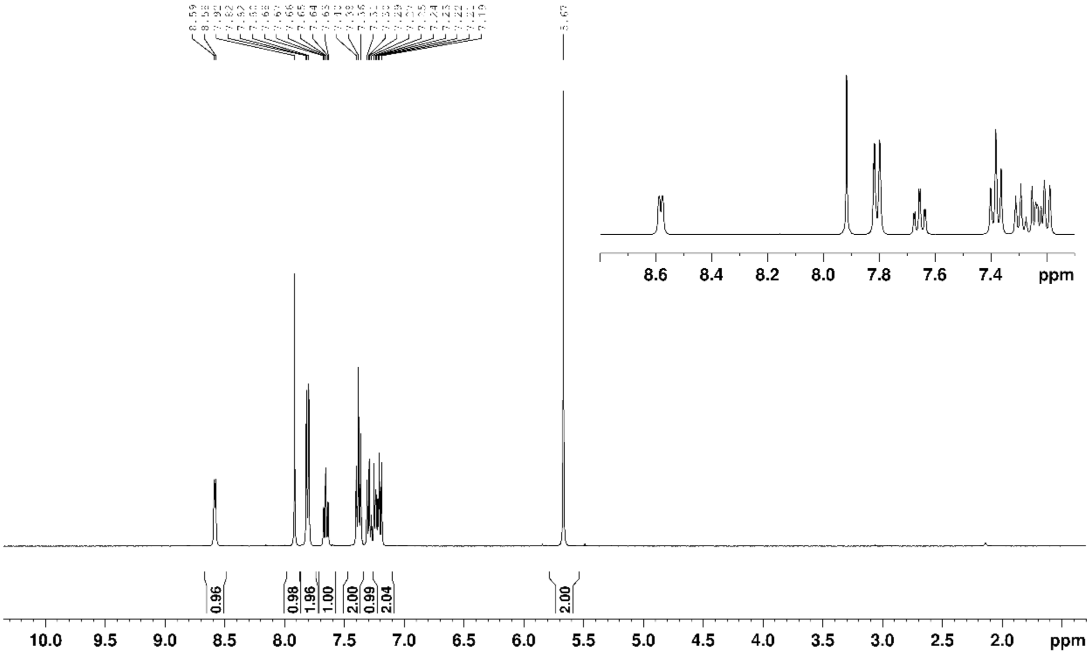

Figure S1. ^{1}H NMR spectrum (400 MHz, $CDCl_3$) of the ligand pictz.

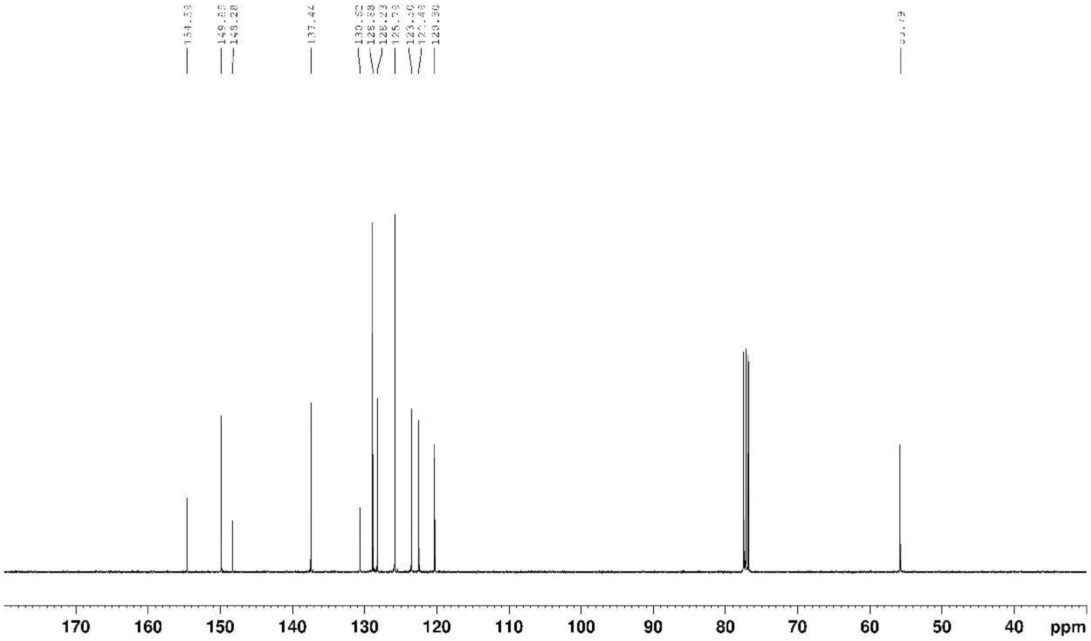

Figure S2. ^{13}C NMR spectrum (101 MHz, $CDCl_3$) of the ligand pictz.

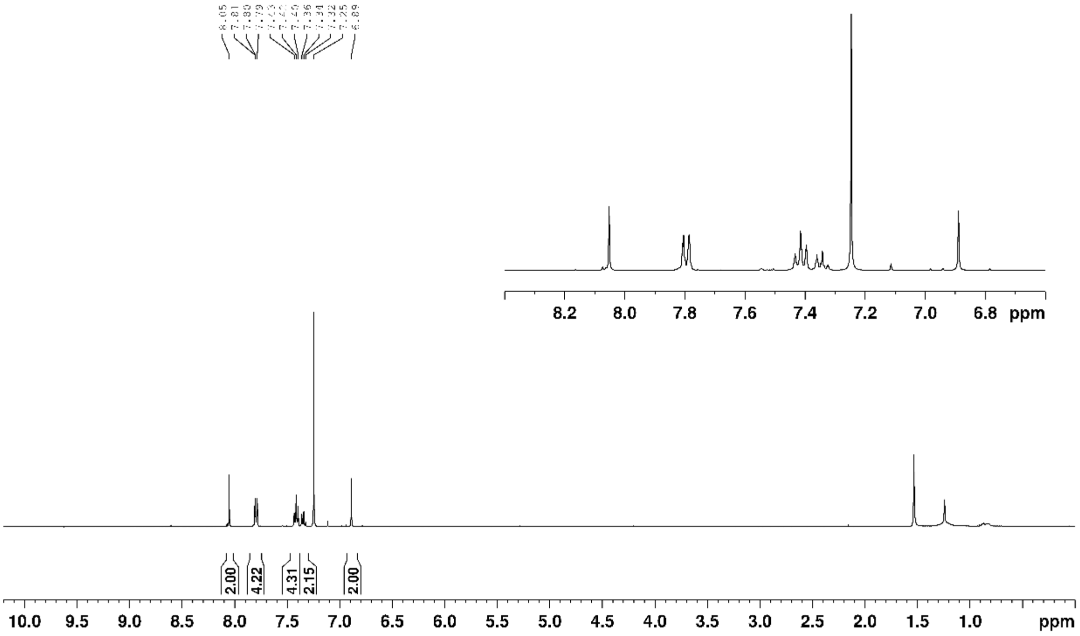

Figure S3. ^{1}H NMR spectrum (400 MHz, $CDCl_3$) of btzm.

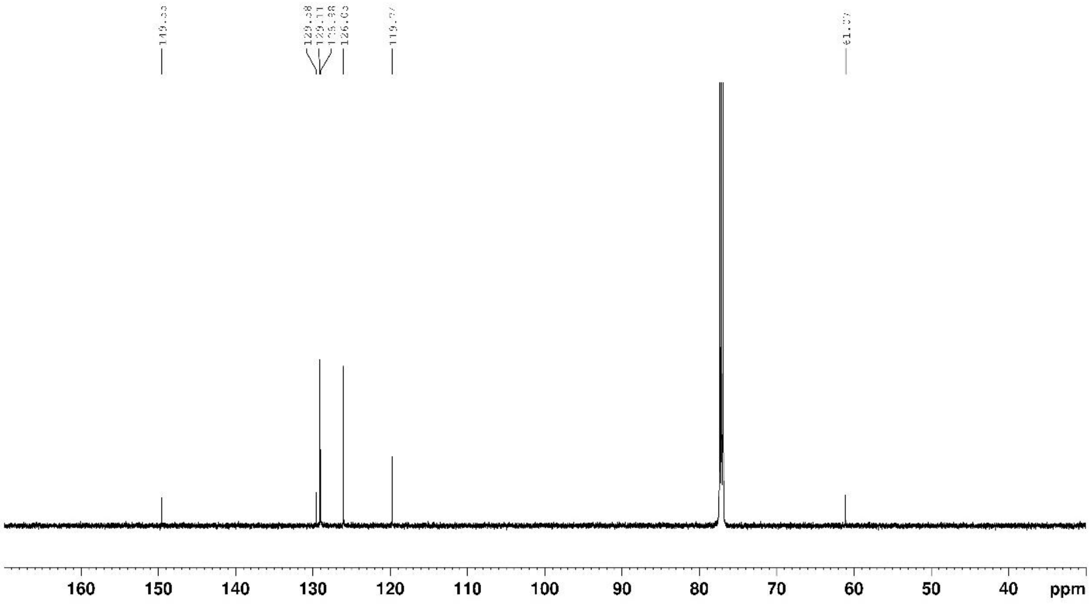

Figure S4. ^{13}C NMR spectrum (151 MHz, $CDCl_3$) of btzm.

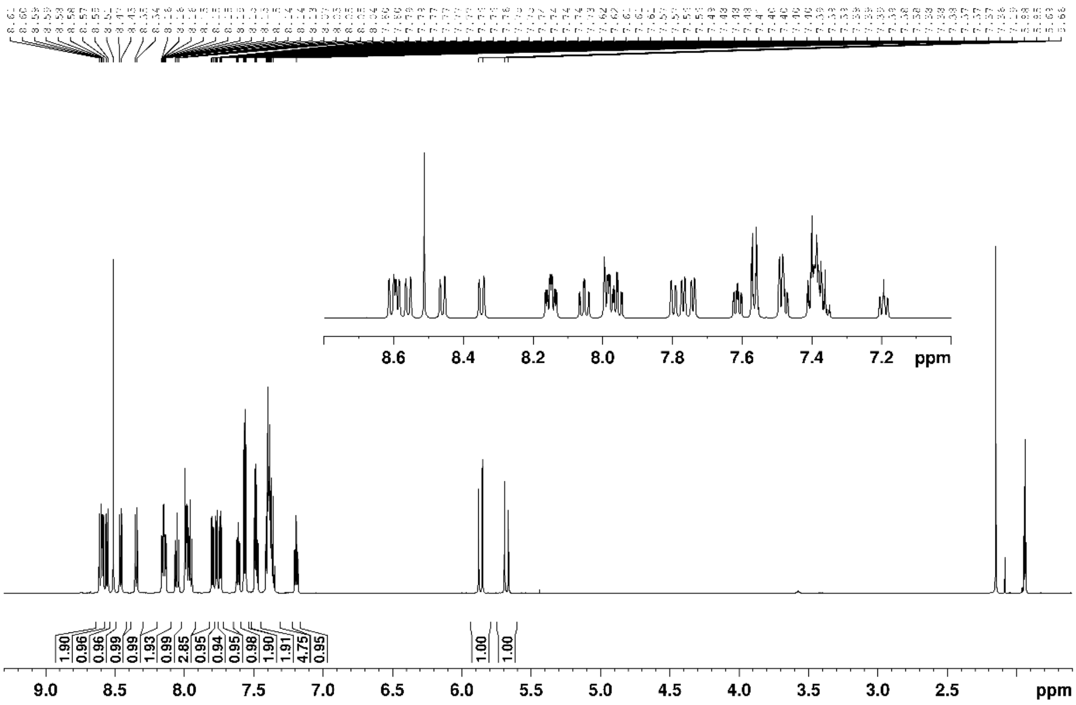

Figure S5. ^{1}H NMR spectrum (600 MHz, d_3-MeCN) of $[Ru(bpy)_2(pictz)]^{2+}$ (**1**).

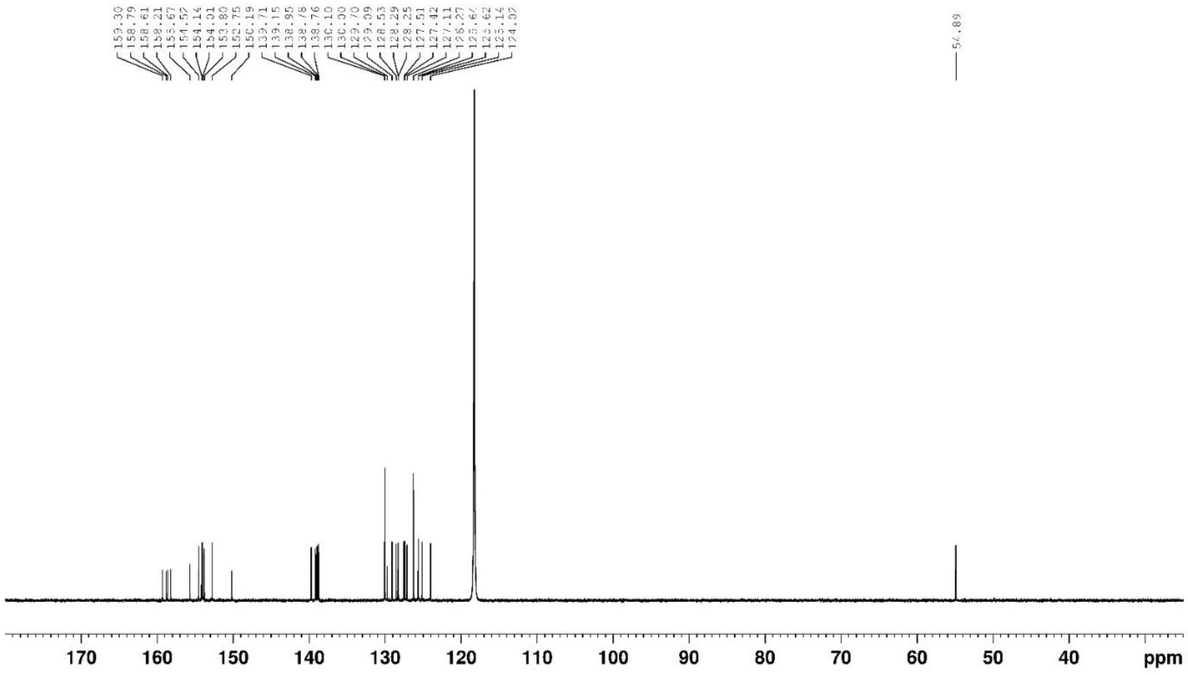

Figure S6. ^{13}C NMR spectrum (151 MHz, d_3-MeCN) of $[Ru(bpy)_2(pictz)]^{2+}$ (**1**).

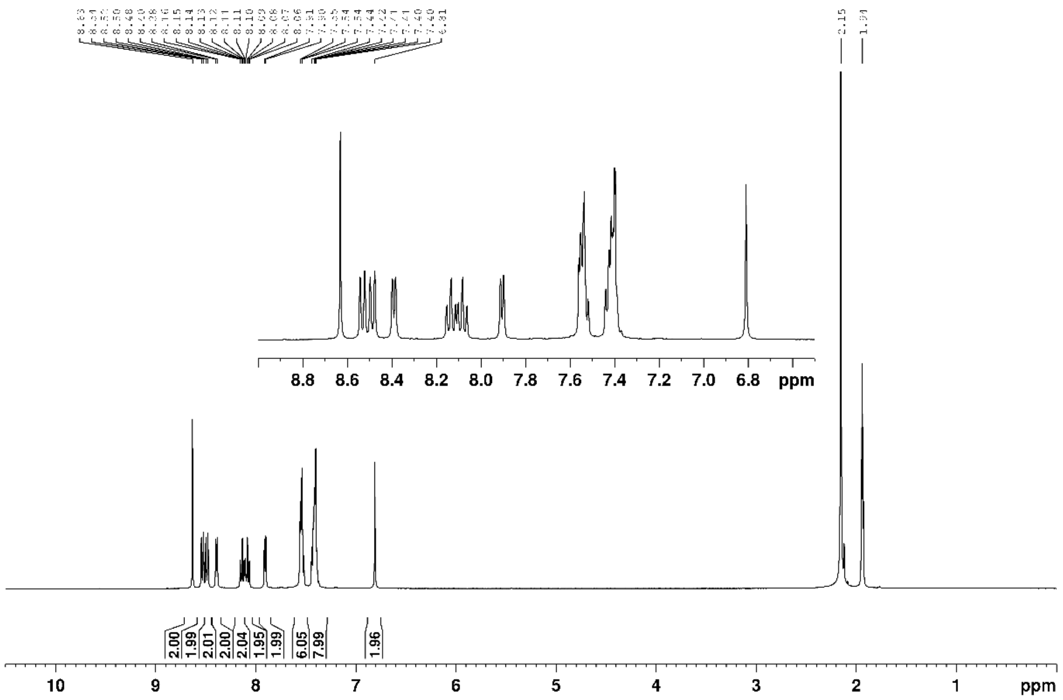

Figure S7. ^{1}H NMR spectrum (400 MHz, d_3-MeCN) of $[Ru(bpy)_2(btzm)]^{2+}$ (**2**).

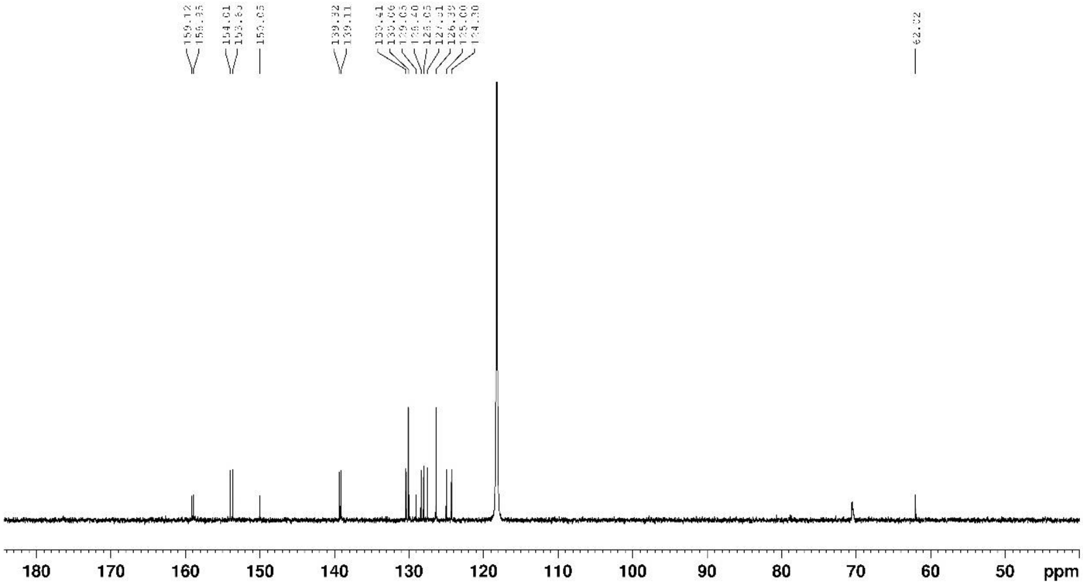

Figure S8. ^{13}C NMR spectrum (151 MHz, d_3-MeCN) of $[Ru(bpy)_2(btzm)]^{2+}$ (**2**).

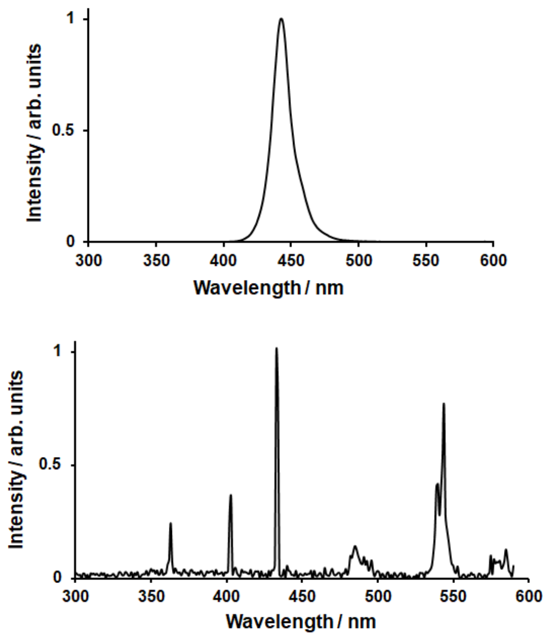

Figure S9. Normalised spectra of output from the 446 nm LED (left) and 23 W fluorescent lamp (right) used in photolysis experiments.

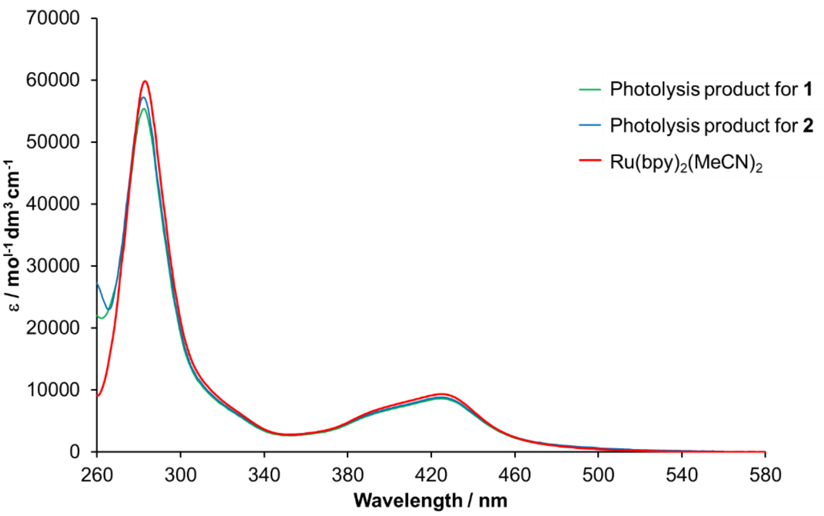

Figure S10. UV-vis absorption spectra after completion of photolysis (λ^{ex} = 446 nm) of complexes **1** and **2** with comparison to spectrum of an authentic sample of $[Ru(bpy)_2(NCMe)_2]^{2+}$.

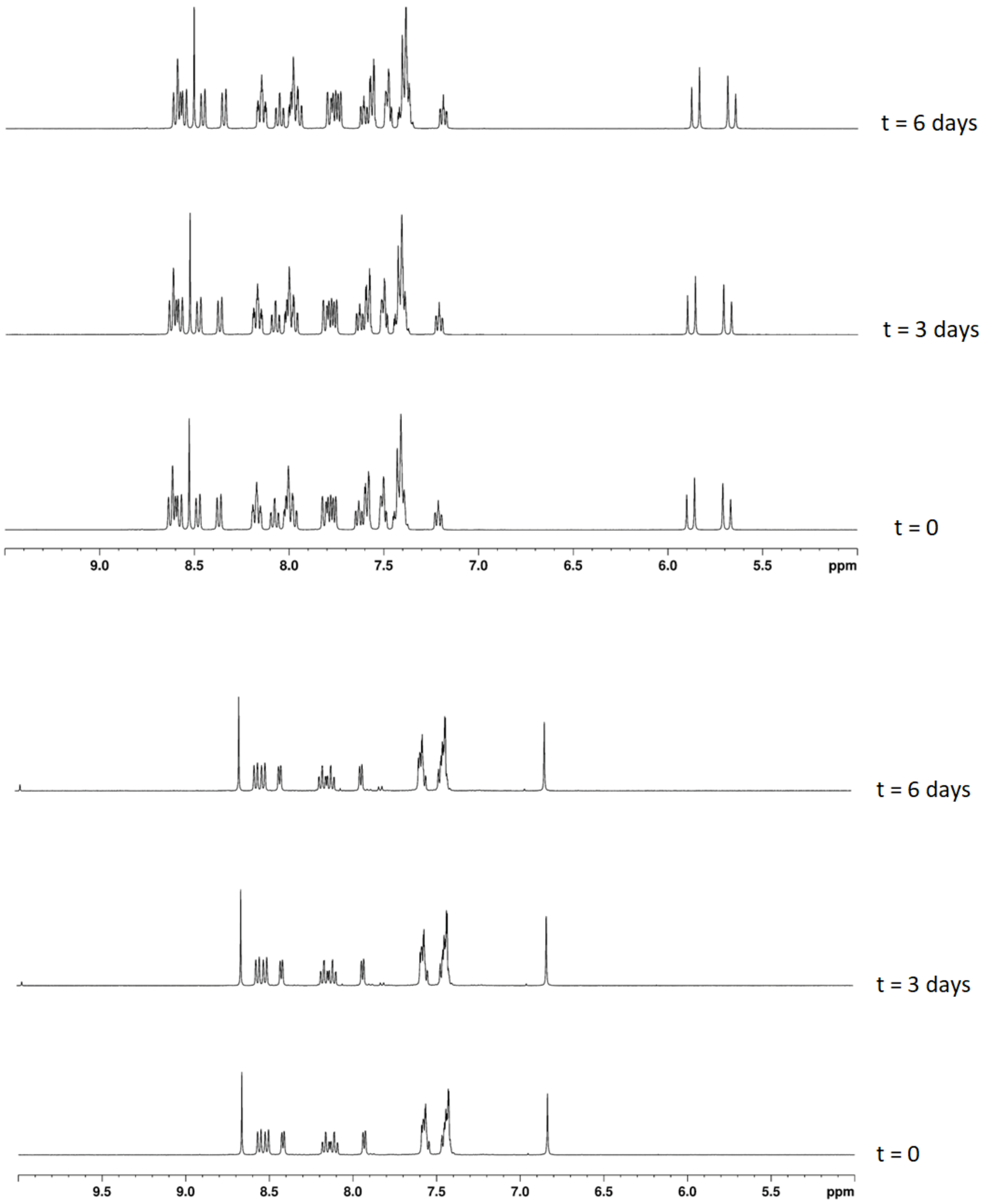

Figure S11. ^{1}H NMR spectra of **1** (top) and **2** (bottom) in d_3-acetonitrile. Samples left in the dark to test thermal stability.

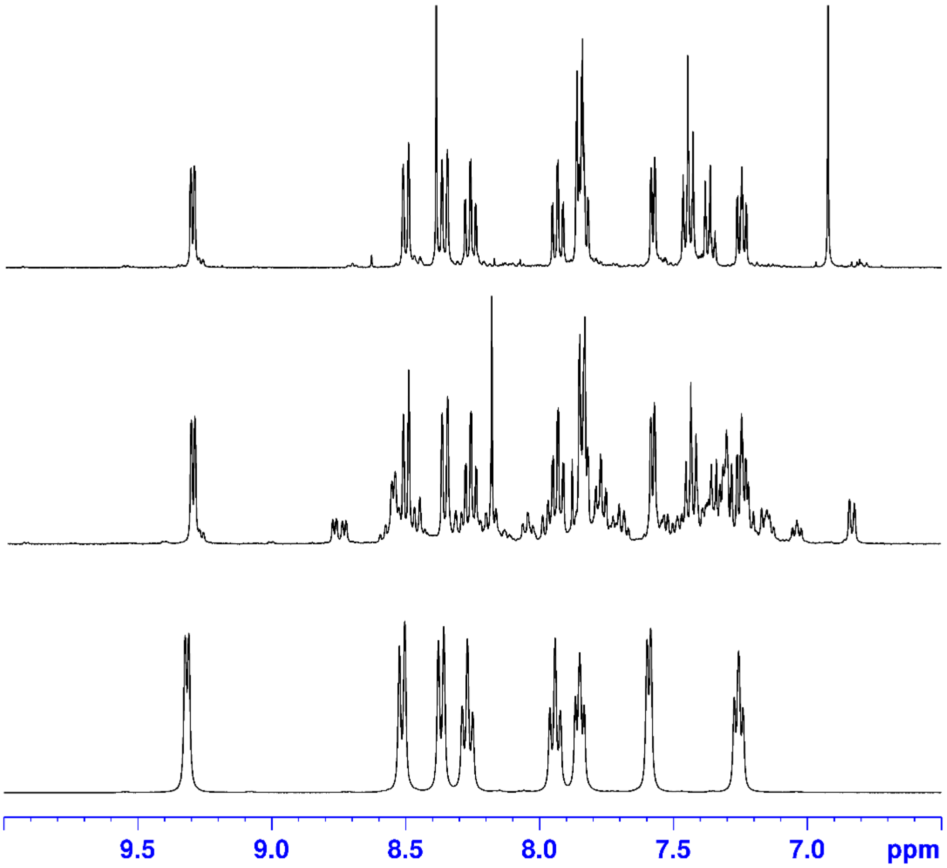

Figure S12. ^{1}H NMR spectra of an authentic sample of $[Ru(bpy)_2(NCMe)_2]^{2+}$ (bottom) and spectra showing its formation as a result of photolysis of **1** (middle) and **2** (top). All spectra recorded in d_3-acetonitrile.

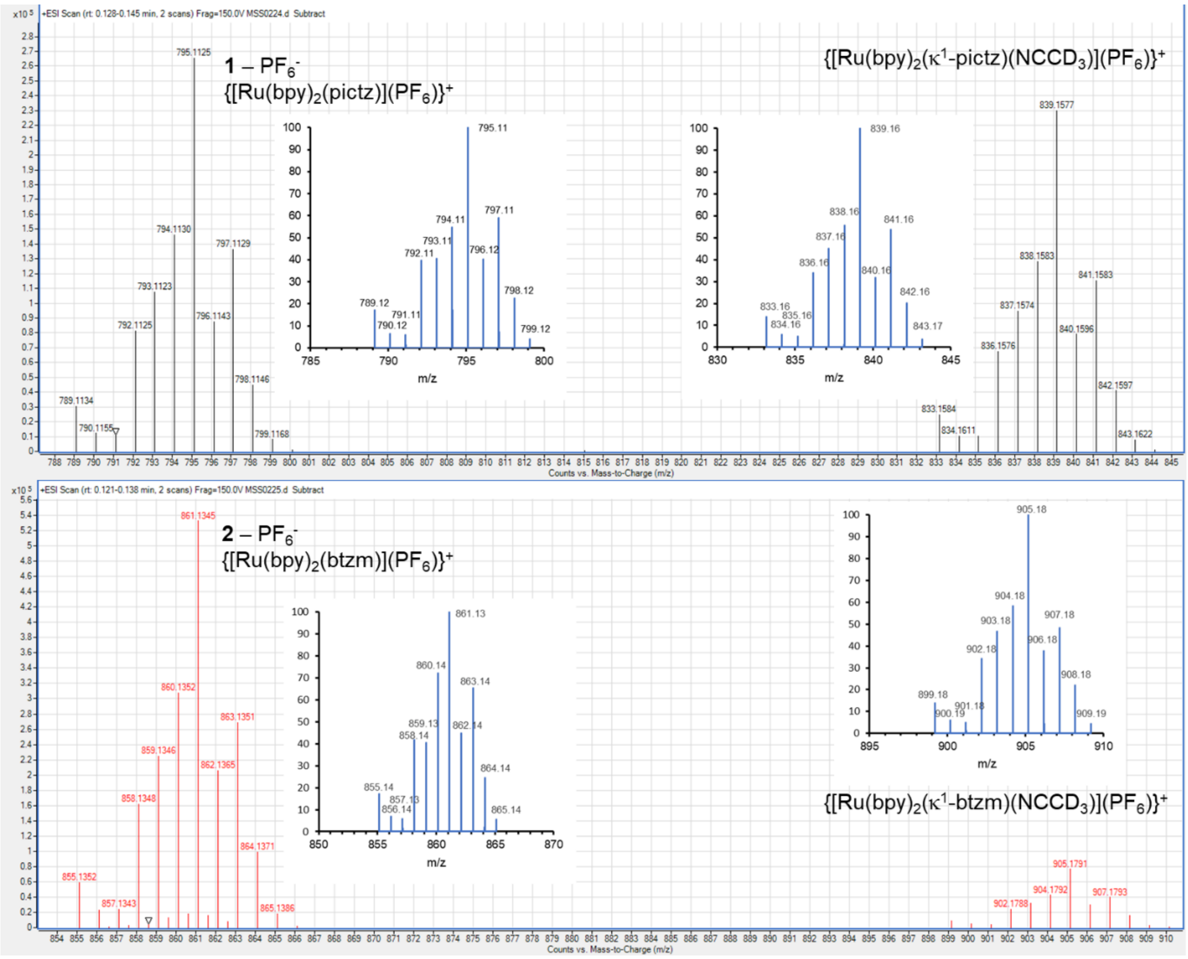

Figure S13. Electrospray mass spectra for samples of **1** (top) and **2** (bottom) in d_3-acetonitrile mid-photolysis showing the ion-pairs $\{[Ru(bpy)_2(pictz)](PF_6)\}^+$ and $\{[Ru(bpy)_2(pictz)](PF_6)\}^+$ and their photochemical ligand-loss intermediates $\{[Ru(bpy)_2(\kappa^1\text{-pictz})(NCCD_3)](PF_6)\}^+$ and $\{[Ru(bpy)_2(\kappa^1\text{pictz})(NCCD_3)](PF_6)\}^+$ respectively (insets are predicted spectra for ions shown). The light source used for photolysis was a 23 W domestic fluorescent light bulb (Hg emission lines).

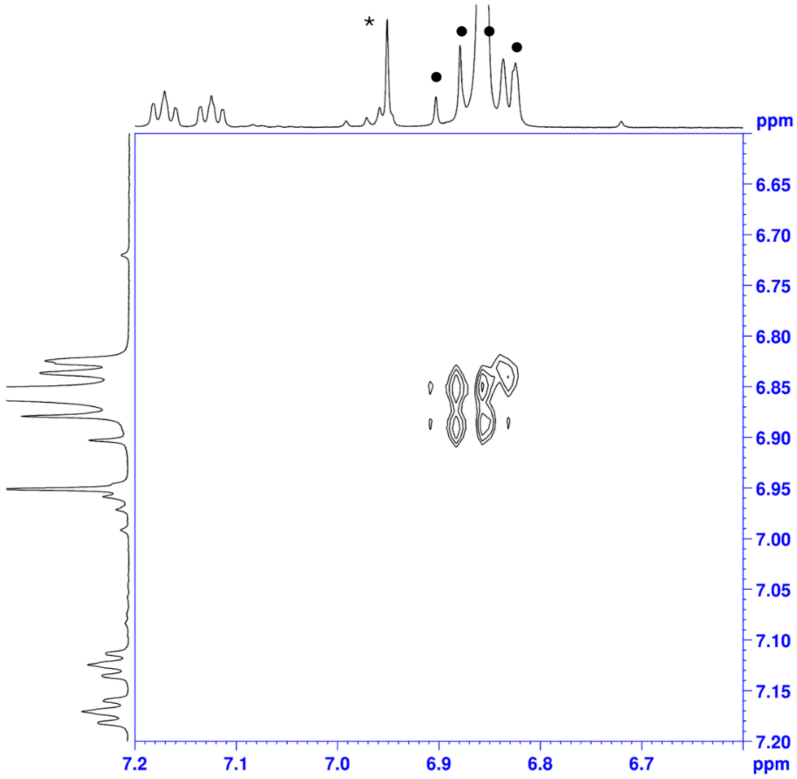

Figure S14. ^{1}H-^{1}H COSY spectrum of the region for the methylene protons of **2** after partial photolysis in d_3-acetonitrile showing correlation of signals for diasteretopic CH_2 protons of proposed ligand-loss intermediate **5-tz$^{(N3)}$**.

Stability studies of intermediate photoproducts

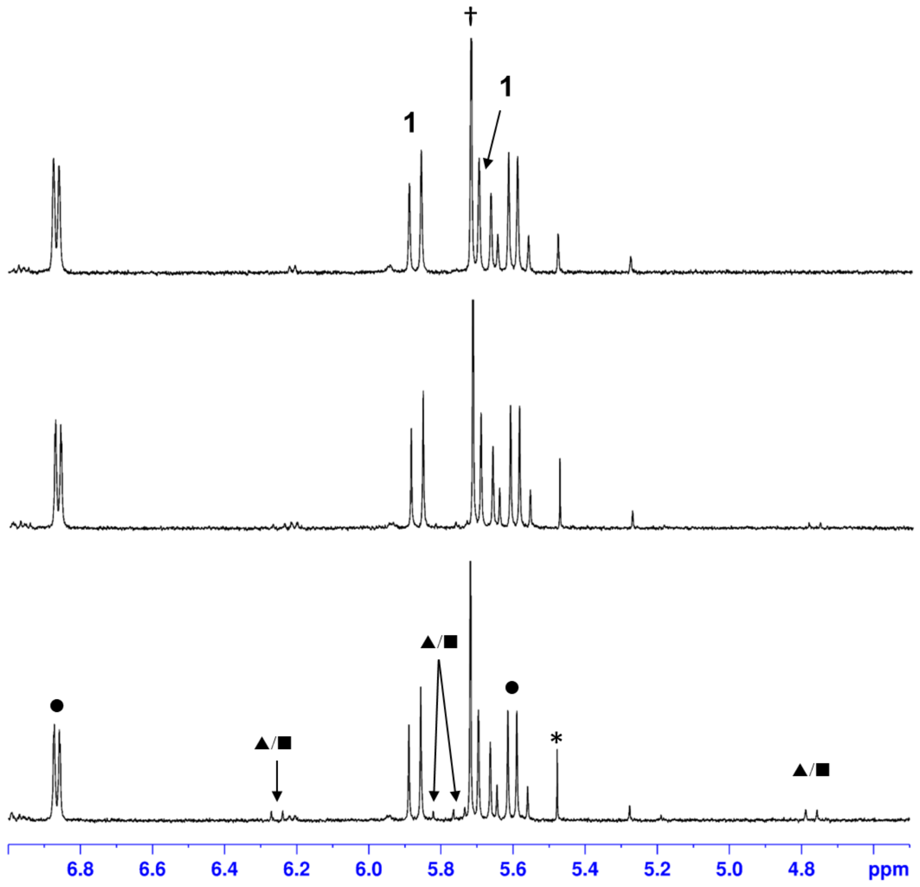

Figure S15 ^{1}H NMR spectra of a partially photolyzed sample of **1** showing changes in upon storing in the dark for **1**, **4-tz$^{(N3)}$** (●), **4-py** and **4-tz$^{(N2)}$** (▲ & ■), and free pictz ligand (†) (bottom: after 5 minutes photolysis; middle: after 1½ hours in the dark; top: sample left overnight in the dark; * solvent impurity). The light source used for photolysis was a 23 W domestic fluorescent light bulb (Hg emission lines).

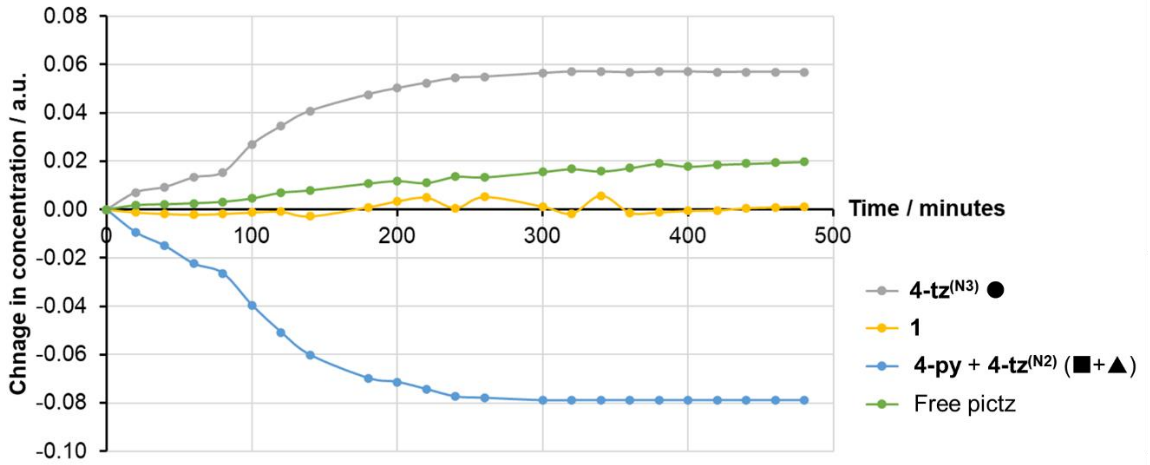

Figure S16. Change in concentrations for **1**, **4-tz$^{(N3)}$**, combined **4-py** & **4-tz$^{(N2)}$** and free pictz ligand for a partially photolyzed NMR sample of **1**. Sample was left in the dark in the bore of the spectrometer with spectra recorded at 20 minutes intervals. The light source used for photolysis was a 23 W domestic fluorescent light bulb (Hg emission lines).

***In-situ* real-time kinetic studies of photochemical reactivity by NMR and fitting to the model.**

Photochemical reactivity of 1.

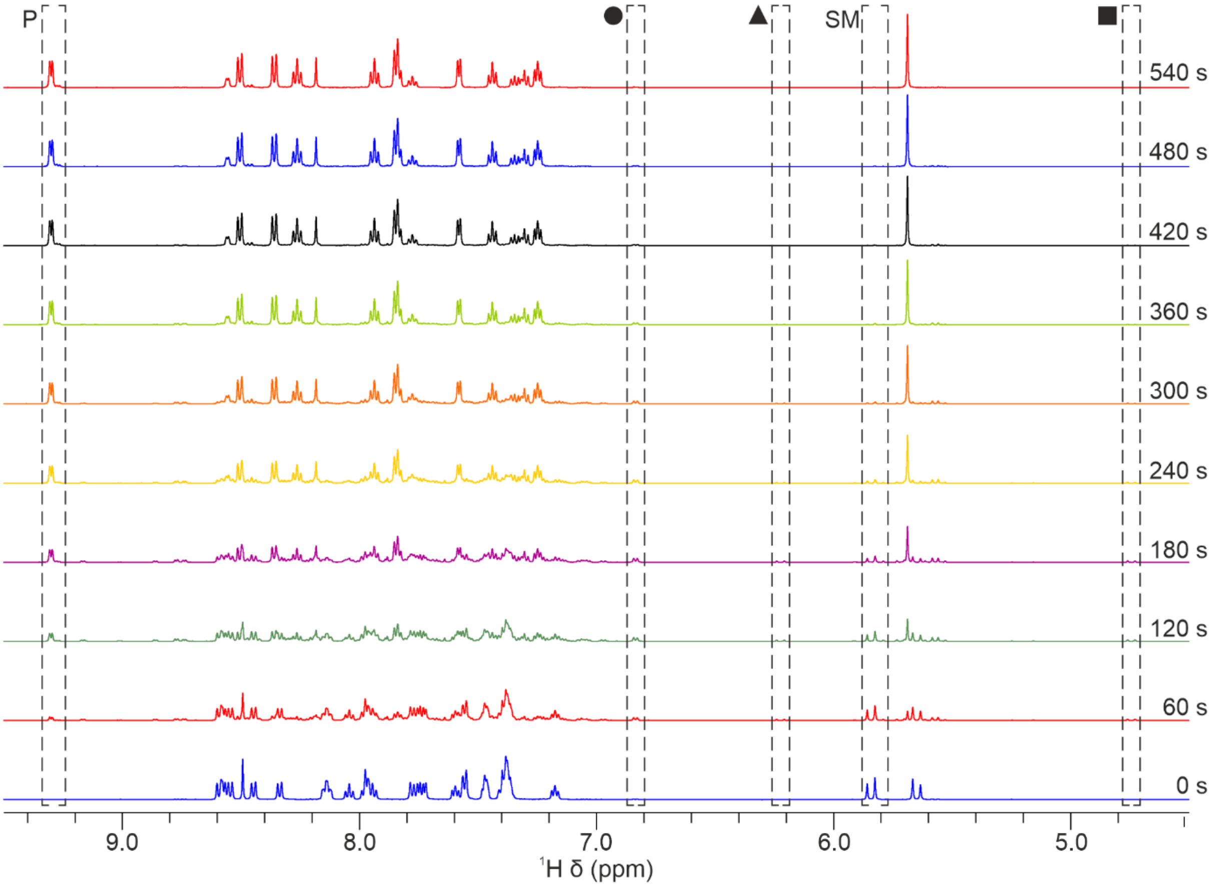

Figure S17. ^{1}H NMR spectra recorded during NMRtorch illumination (λ^{ex} = 459 nm) of Complex **1**. The sample was irradiated with a 2 s pulse prior to each spectral acquisition. For the selected spectra are taken after successive 60 s cumulative irradiation periods with signals used in kinetic analyses highlighted (starting material **1** (labelled "SM"), photoproduct $[Ru(bpy)_2(NMCe)_2]^{2+}$ (labelled "P") and intermediates **4-tz$^{(N3)}$** (labelled ●) and **4-py/4-tz$^{(N2)}$** (labelled ▲/■). Starting material integral SM was corrected to remove contribution from overlapping signal from intermediate ▲.)

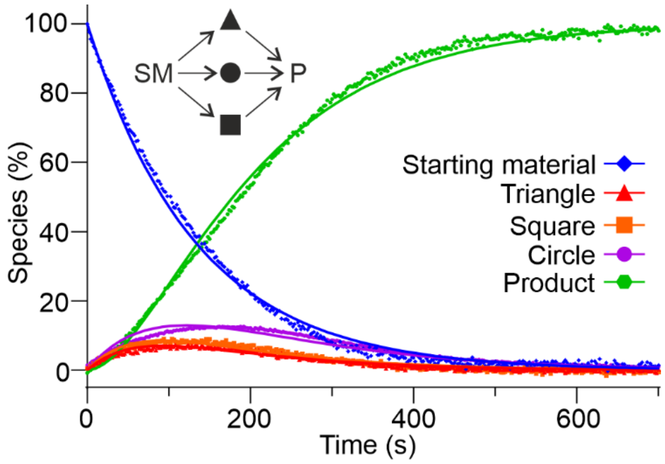

Figure S18. Kinetics of complex **1** photolysis studied by in situ photo-NMR (λ^{ex} = 459 nm). Experimental data fitted to kinetic model using DynaFit 4.

Modelling of photochemical conversion of complex 1.

General scheme with ▲ (“T” in table below) linked to ● (**4-tz^(N3)**, “C” in table below):

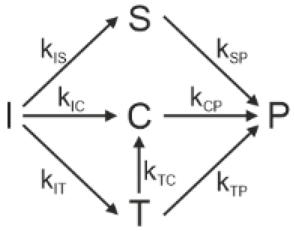

Model Ranking	Model	k_{IS}	k_{IC}	k_{IT}	k_{SP}	k_{CP}	k_{TP}	k_{TC}	SSQ	DeltaAIC
1 Direct+Indirect	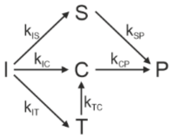	3.11 ± 0.06	1.94 ± 0.10	2.44 ± 0.08	18.51 ± 0.37	14.00 ± 0.21	X	17.33 ± 0.63	2509.54	0
2 Direct+Indirect	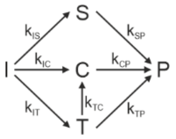	3.08 ± 0.10	1.96 ± 0.11	2.46 ± 0.10	18.25 ± 0.63	13.76 ± 0.56	0.81 ± 1.69	16.69 ± 1.47	2509.07	1.7
3 Direct	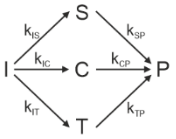	2.53 ± 0.07	2.92 ± 0.05	2.06 ± 0.07	14.84 ± 0.47	9.14 ± 0.17	14.59 ± 0.57	X	2806.37	196.2
4 Indirect	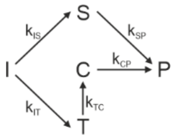	3.50 ± 0.05	X	3.95 ± 0.05	20.85 ± 0.35	12.89 ± 0.19	X	29.02 ± 0.54	2962.51	289.2
5 Indirect+(Direct)	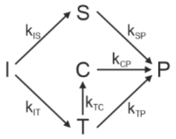	3.50 ± 0.05	X	3.95 ± 0.05	20.85 ± 0.35	12.89 ± 0.19	0.00 ± 1.35	29.02 ± 0.54	2962.52	291.2

(Rates x 10^{-3} % s^{-1})

General scheme with ■ ("S" in table below) linked to ● (**4-tz**$^{\mathbf{(N3)}}$, "C" in table below):

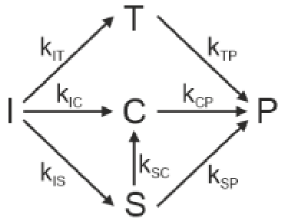

Model Rank	Model	k_{IT}	k_{IC}	k_{IS}	k_{TP}	k_{CP}	k_{SP}	k_{SC}	SSQ	DeltaAIC
1<br>Direct+Indirect	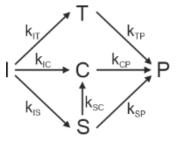	2.59 ± 0.10	1.95 ± 0.11	2.96 ± 0.09	18.61 ± 0.78	13.83 ± 0.54	3.37 ± 1.38	14.07 ± 1.24	2508.02	0
2<br>Direct+Indirect	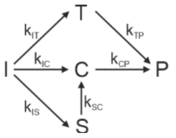	2.80 ± 0.06	1.84 ± 0.10	2.86 ± 0.08	20.24 ± 0.46	15.01 ± 0.22	X	16.82 ± 0.51	2518.00	5.0
3<br>Direct	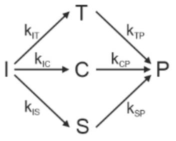	2.06 ± 0.07	2.92 ± 0.05	2.53 ± 0.07	14.59 ± 0.57	9.14 ± 0.17	14.84 ± 0.47	X	2806.47	195.2
4<br>Indirect	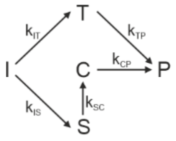	3.27 ± 0.05	X	4.18 ± 0.05	23.80 ± 0.44	13.70 ± 0.19	X	25.12 ± 0.40	2961.60	287.7
5<br>Indirect+(Direct)	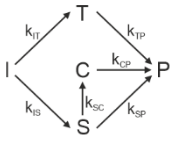	3.27 ± 0.05	X	4.18 ± 0.05	23.80 ± 1.01	13.70 ± 0.39	0.00 ± 1.30	25.12 ± 0.71	2961.61	289.7

(Rates x 10^{-3} % s^{-1})

Irrespective of the position of S and T in the initial model, models with Direct+Indirect formation of C (**4-tz**$^{\mathbf{(N3)}}$) from the initial starting material **1** (ie, Models 1 and 2 in both tables above) fit the experimental data better than models with Indirect Only formation of C from T/S, or Direct Only models.

The remaining scenarios (see below, S is interchangeable with T) are difficult to distinguish. However, it can be noted that in any case there appear to be only two major intermediates for the last step of this photoreaction, not three (as conversion of the third intermediate to P appear to be much slower than for the other two). "Last step intermediates" therefore here are C (i.e., molecules with characteristic signals marked with circles) and either S or T. Notably, C is produced both directly (from **1**), and indirectly (from either S or T). Interestingly, the rate constants appear to be very similar overall regardless of the S vs T assignment, with initial conversions slower than the final stem conversions.

The summary of the most likely scenarios is as follows (rates x 10^{-3} % s^{-1}):

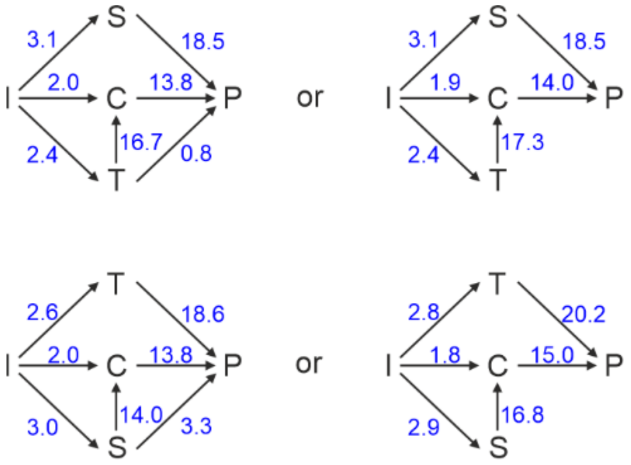

Photochemical reactivity of 2.

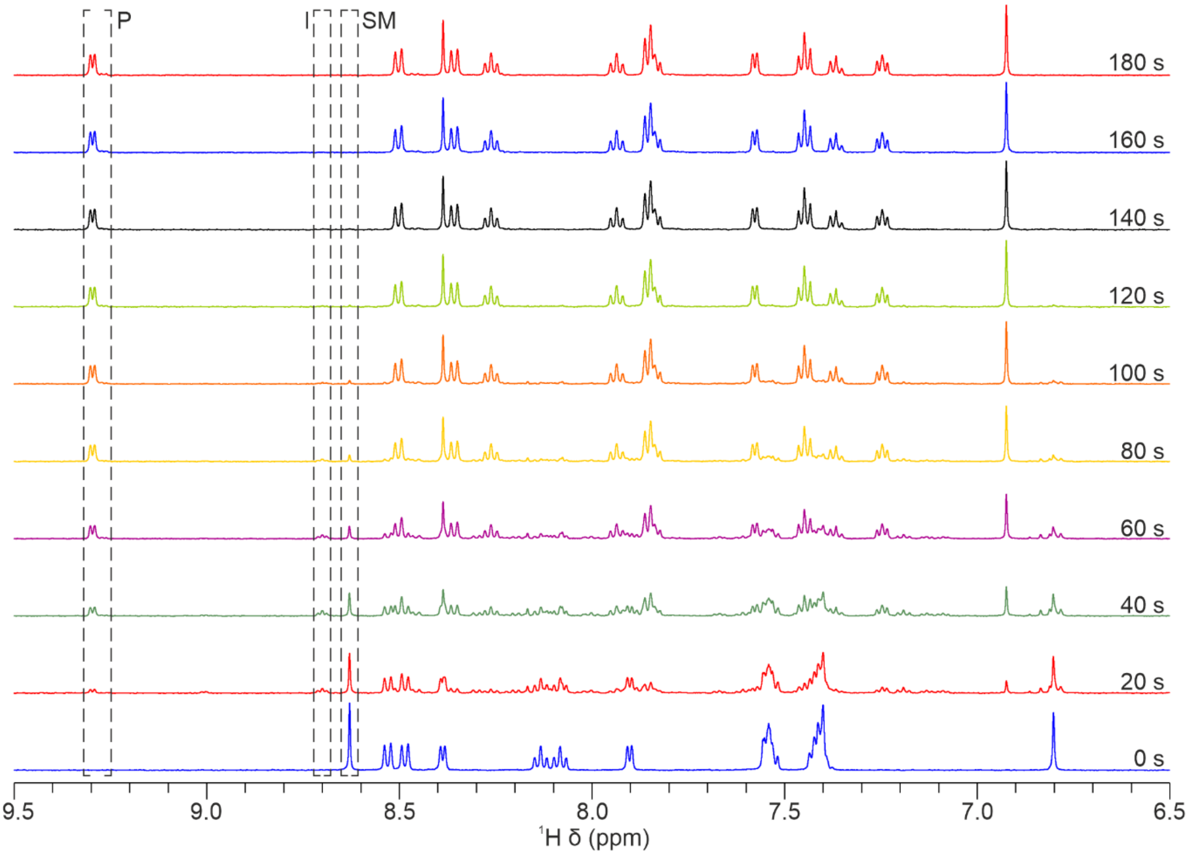

Figure S19. ^{1}H NMR spectra recorded during illumination of complex **2** (λ^{ex} = 459 nm). The sample was irradiated with a 2 s pulse prior to each spectral acquisition. Selected spectra taken after successive 20 s cumulative irradiation periods with signals used for kinetic analyses highlighted (starting material **2** (labelled "SM"), photoproduct **3** (labelled "P") and intermediate **5** (labelled "I")).

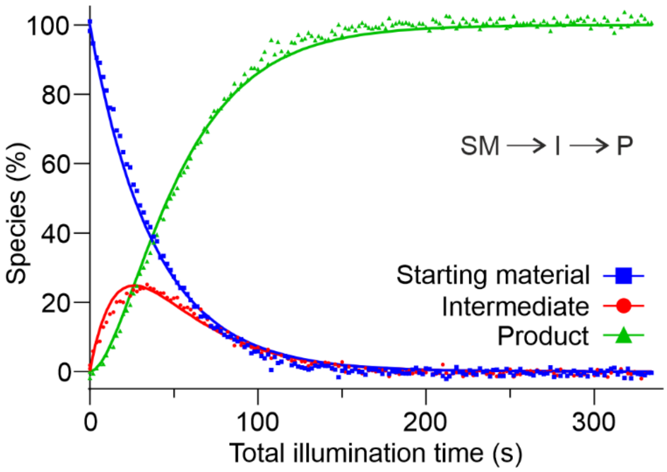

Figure S20. Kinetics of complex **2** photolysis studied by *in situ* photo-NMR (λ^{ex} = 459 nm). Experimental data fitted to kinetic model using DynaFit 4.

Dynafit model:

SM → I : k1

I → P : k2

Rates:

	k, SM → I (% s^{-1})	k, I → P (% s^{-1})
Complex **2**	0.02621 ± 0.00016	0.05300 ± 0.00063
